# Genome Mining and Molecular Networking-Targeted Discovery of Siderophores with Plant Growth-Promoting Activities from the Marine-Derived *Streptomonospora nanhaiensis* 12A09^T^

**DOI:** 10.3390/md24010007

**Published:** 2025-12-22

**Authors:** Yan Bai, Weixian Gao, Wendian Zhao, Amr A. Arishi, Zhuo Shang, Jiangchun Hu, Huaqi Pan

**Affiliations:** 1 CAS Key Laboratory of Forest Ecology and Silviculture, Institute of Applied Ecology, Chinese Academy of Sciences, Shenyang 110016, China; baiy@iae.ac.cn (Y.B.); gaoweixian20@mails.ucas.ac.cn (W.G.); zwd5613@163.com (W.Z.); hujc@iae.ac.cn (J.H.); 2 School of Molecular Sciences, The University of Western Australia, Perth, WA 6009, Australia; amr.arishi@research.uwa.edu.au; 3 School of Pharmaceutical Science, Shandong University, Jinan 250012, China; zshang@sdu.edu.cn

**Keywords:** siderophores, desferrioxamine, genome mining, molecular networking, plant growth-promoting activity

## Abstract

Plant growth regulators (PGRs) significantly contribute to enhancing crop quality and yield. There is an urgent market demand for innovative natural PGRs. Marine natural products have the potential to serve as valuable sources of PGRs. To discover natural siderophore-type PGRs from marine natural products, according to a systematic pipeline for efficient lead-structure discovery from microbial natural products (SPLSD), a unique desferrioxamine-like siderophore biosynthetic gene cluster was discovered and activated by genome mining and culture regulation from a novel species, *Streptomonospora nanhaiensis* 12A09^T^. Some potentially new desferrioxamine derivatives were further discovered by the LC-MS/MS molecular network. Three new desferrioxamine derivatives, desferrioxamines C1, C2, and G3 (**1**–**3**) and three known ones, terragine E (**4**) and desferrioxamines E and D2 (**5**–**6**), were selectively isolated and identified using chromatography and spectroscopy techniques from *S. nanhaiensis* 12A09^T^. In the ferric iron-chelating assay, **4** and **5** showed moderate Fe (III)-complexing capability, compared with desferrioxamine mesylate. In the plant growth-regulatory assay, **1**, **5**, and **6** potently boosted the root length of *Oryza sativa* and *Brassica campestris* seedlings, equivalent to gibberellin. This study reports the first discovery of desferrioxamine derivatives exhibiting plant growth-promoting activity. These findings offer valuable lead compounds for PGRs.

## 1. Introduction

The global human population is estimated to increase to approximately 9.7 billion by 2050, representing an increase of over 1.8 billion compared to the current population [1]. To meet the growing demand for food, there must be a significant improvement in crop productivity. Plant growth regulators (PGRs) significantly contribute to enhancing crop quality and yield and to improving agricultural practices [2]. The systematic use of PGRs, such as ethylene and acetylene, began in the 1930s [2]. Since then, many natural PGRs that promote plant growth have been discovered, including gibberellin (GA), auxins, abscisic acid, brassinosteroids, cytokinins, and jasmonic acid [2,3]. The global market for PGRs has experienced substantial growth, with sales increasing from approximately USD 1.5 billion in 2015 to roughly USD 2–3 billion in 2022 [4]. However, it is worth noting that PGRs primarily influence the growth of fruits and stems, and relatively few have been developed that specifically target root growth [4]. Thus, the demand for root-promoting PGRs has garnered significant scientific attention.

Marine natural products have garnered significant attention in recent years owing to their remarkable structural diversity and potent biological activities [5,6]. Although marine natural products have been extensively studied as insecticides and bactericides [7,8], surprisingly few reports exist regarding their utility as PGRs. To date, only alginate oligosaccharides (AOSs), the degradation products of alginate, have been demonstrated to enhance root growth [9]. The above reports indicate that, despite the structural diversity of marine natural products, their PGR functions have not been adequately investigated. Thus, the discovery of new natural PGRs targeting root growth from marine natural products can provide new opportunities for enhancing crop quality and yield.

Iron plays a critical role as a vital element for all living organisms. Iron deficiency can disrupt critical physiological processes, while iron overload can result in cellular dysfunction and compromised organ functions, potentially undermining overall health [10]. Siderophores (molecular weight < 1500 Da) are one type of iron-acquisition system employed to capture iron [11]. Siderophores possess a high affinity for Fe^3+^, enabling them to convert insoluble iron into soluble forms and form highly stable compounds with iron [12]. Several studies have shown that plant growth-promoting rhizobacteria (PGPR) can produce siderophores, which play an important role in plant growth [13]. Some research has reported that siderophore-containing extracts enhance iron availability, alleviate plant chlorosis, and promote plant growth [14,15,16]. However, no studies have yet demonstrated that pure siderophores alone can promote plant growth.

Additionally, the discovery of microbial lead compounds currently confronts several critical challenges. (1) The increasing redundancy in strain research raises the core question of how to optimally screen and select target strains. (2) Whole-genome sequencing is so costly that genomic mining cannot be performed for every strain. (3) Efficient recognition of metabolites derived from activated biosynthetic gene clusters (BGCs) remains difficult. (4) Many novel natural products fail to exhibit detectable bioactivity in screening assays. To address these issues, we used a systematic and highly predictive integrated strategy, as reported in [17], which was designated SPLSD (a systematic pipeline for efficient lead-structure discovery from microbial natural products) based on the selection of promising strains and multi-omics mining (Figure S1). The workflow comprises the following steps. Firstly, a taxonomy-guided approach is employed to select promising strains. Reference genomes from closely related strains available in public databases are employed for in silico mining of target BGCs, enabling the prioritization of candidates, which are subsequently validated through whole-genome sequencing and bioinformatic analysis. Secondly, a combination of specific and non-specific activation methods is applied to induce BGC expression in the selected strains, thereby expanding metabolic diversity. This recognition step is coupled with metabolomic profiling, multiple bioactivity screening assays, and transcriptome analysis to predict the structural features, novelty, and potential bioactivities of the activated metabolites. Finally, structure- and activity-guided isolation strategies are used to rapidly purify and characterize novel bioactive lead compounds.

**Figure 1:**
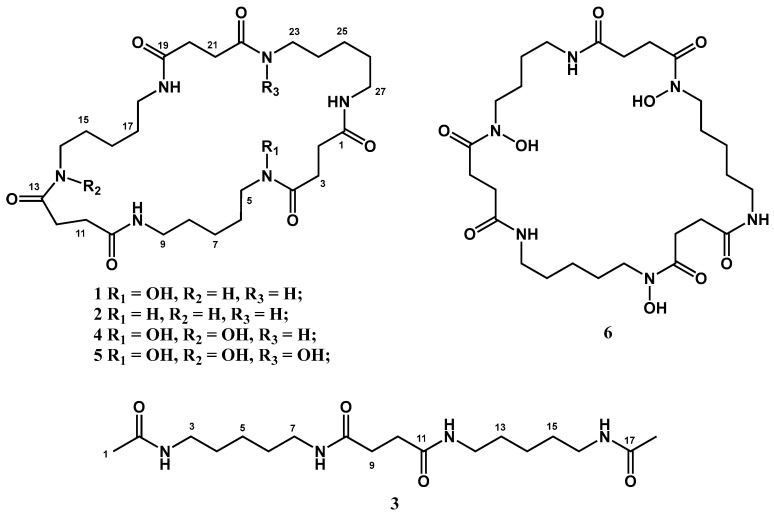
The structures of compounds **1**–**6**.

To discover natural siderophore-type PGRs, according to SPLSD, we performed genome mining on fifty-six sequenced genomes that we previously obtained. The marine-derived *Streptomonospora nanhaiensis* 12A09^T^ has attracted our attention due to the special deferoxamine-like BCG. Based on the culture regulation approach, the deferoxamine-like BGC was activated by NO2 medium, and its extracts showed significant ferric iron-chelating activity. Herein, the chemical components of *S. nanhaiensis* 12A09^T^ extracts are investigated, guided by molecular networking and the ferric iron-chelating assay, resulting in the identification of six siderophores (**1**–**6**), including three new compounds, designated desferrioxamines C1, C2, and G3 (**1**–**3**) (Figure 1). The ferric iron-chelating and plant growth-promoting activities of **1**–**6** were evaluated.

## 2. Results and Discussion

### 2.1. Complete Genome Features of S. Nanhaiensis 12A09^T^

**Figure 2:**
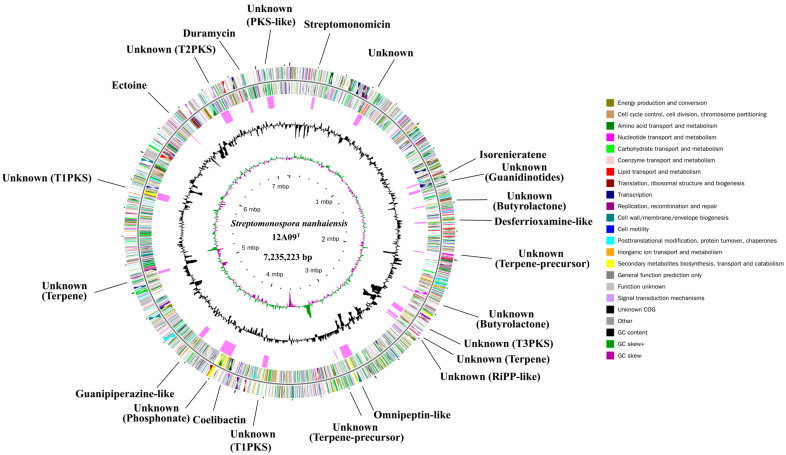
The chromosome genome of *S. nanhaiensis* 12A09^T^. The genome map is composed of six circles. From the inner to outer circles, each circle displays information regarding the genome. (1) Scale (bp); (2) GC skew; (3) GC content; (4) predictive secondary metabolite clusters; (5) and (6) the COG of each CDs.

The complete genome comprises a 7,235,223-bp circular chromosome with 73.0% GC content (Figure 2) and a 35,208-bp circular plasmid. The predicted chromosome genome consists of 6200 ORFs, comprising five copies of 16S-23S-5S rRNA and 59 tRNA. Through querying the COG database [18], 5384 protein-coding genes were successfully annotated and subsequently categorized into four primary functional classifications (Table S1). The most highly represented category was proteins related to metabolic functions (35.4%). The chromosome and plasmid genome sequence for *S. nanhaiensis* 12A09^T^ were deposited in the GenBank database and were accessible under the accession numbers CP113264 and CP113265, respectively.

### 2.2. Genome Mining of BGCs

The genome of *S. nanhaiensis* 12A09^T^ was analyzed via antiSMASH 8.0, followed by manual validation (Figure 2 and Table 1). A total of 23 BGCs were proposed for this strain, occupying 9.7% of the chromosome. Among these BGCs, six encode polyketide synthases (PKS) (unknown); five encode terpene biosynthesis (isorenieratene et al.); three encode nonribosomal peptides (NRPs) (omnipeptin-like, coelibactin, and guanipiperazine-like); two appear to code for butyrolactone (unknown); one is predicted to synthesize siderophores (desferrioxamine-like), along with six other types of BGCs are identified, including those encoding lassopeptide, guanidinotides, RiPP-like, phosphonate, ectoine, and lanthipeptide-class-iii. Notably, more than 65% of the detected BGCs associate with unknown products. Although some BGCs in this strain are known, they are not entirely identical to those reported, such as BGCs 12 and 17 (omnipeptin-like and guanipiperazine-like). These results highlight the strain’s unique genetic characteristics. Therefore, the promising strains selected from unique environments and possessing distinctive taxonomic status represent valuable sources of novel bioactive natural products, constituting a critical first step in the SPLSD strategy [17].

**Figure 3:**
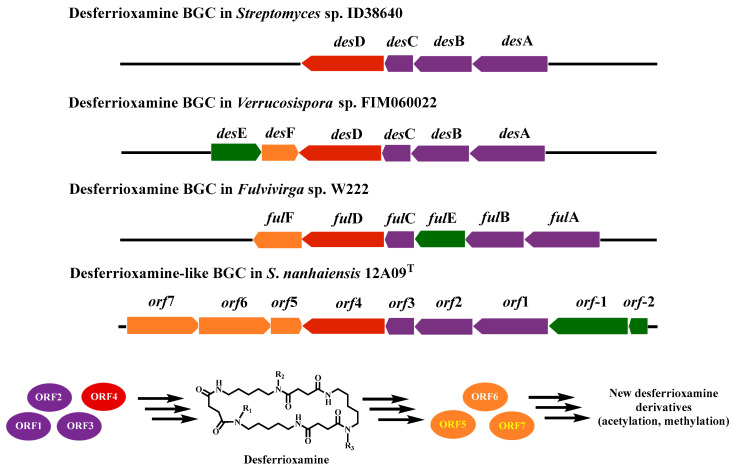
Genetic organization of desferrioxaminamin-related BCGs from different microbes and prediction of desferrioxaminamin derivatives in *S. nanhaiensis* 12A09^T^. ORF1: *L*-2, 4-diaminobutyrate decarboxylase; ORF2: lysine/ornithine *N*-monooxygenase; ORF3: *N*-acetyltransferase; ORF4: siderophore biosynthesis protein; ORF5: methyltransferase; ORF6: acyl-CoA synthetase; ORF7: acyl-CoA transferase.

Among these BGCs, BGC 6 encoded a siderophore that probably synthesized desferrioxamine and its derivatives (Figure 3). It was predicted by the antiSMASH 8.0 that the BGC 6 of strain 12A09^T^ had a high homology with the desferrioxamine BGC of *Streptomyces* sp. ID38640, *Fulvivirga* sp. W222, and *Verrucosispora* sp. FIM060022, respectively [19,20,21]. According to previous reports [12,22], the majority of siderophore classes are biosynthesized by nonribosomal peptide synthetases (NRPSs). However, the biosynthesis of desferrioxamine uses NRPS-independent siderophore pathways (NISs). BGC 6 is composed of seven open reading frames (ORFs). Further analyses using BLAST+2.17.0 and 2ndFind revealed that four proteins encoded by ORFs 1–4 could catalyze the biosynthesis of the desferrioxamine skeleton. In addition, there are three post-modification genes in BGC 6, which encode methyltransferase (ORF5), acyl-CoA synthetase (ORF6), and acyl-CoA transferase (ORF7) (Figure 3 and Table 2). These findings suggest that *S. nanhaiensis* 12A09^T^ is likely capable of producing new desferrioxamine derivatives through post-modification enzymes encoded by BGC 6.

### 2.3. Culture Regulation for Activating the Silent Siderophore BGC

**Figure 4:**
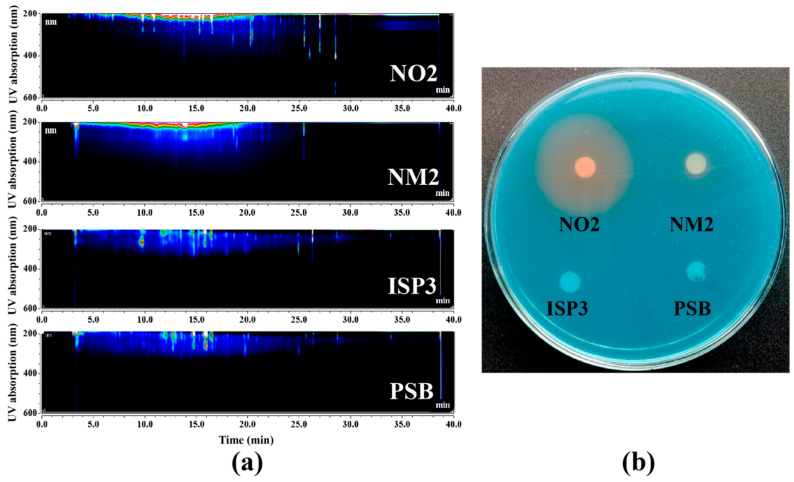
Screening results of culture regulation to activate the siderophore BGC. (**a**) HPLC-DAD analysis of crude extracts from four media of *S. nanhaiensis* 12A09^T^. (**b**) The CAS assay of crude extracts from four media of *S. nanhaiensis* 12A09^T^.

To activate the siderophore BGC 6, four types of media (PSB, NO2, NM2, and ISP3) were employed to culture the strain using a culture-regulation approach. Based on HPLC-DAD analysis, the metabolic fingerprint profiles of crude extracts from NO2 and NM2 media showed significantly different UV absorption from those of ISP3 and PSB media (Figure 4a). In the chrome azurol S (CAS) assay, siderophore production could be judged by observing the size of the distinct visible orange halo. We found that the crude extracts of NO2 and NM2 media both showed a visible orange halo (Figure 4b), suggesting the potential presence of siderophore secondary metabolites. UV absorption at 210 nm (retention time: 5–20 min) further supports this observation. In contrast, the crude extracts from ISP3 and PSB media (UV absorption at 210 and 275 nm with retention time: 10–17 min) showed no ferric iron-chelating activity, indicating an absence of siderophore metabolites. Thus, siderophore BGC 6 could be activated by NO2 or NM2 media. Obviously, the crude extract from the NO2 medium displayed much higher diversity of metabolites and greater ferric iron-chelating activity compared to the NM2 medium crude extract. Thus, the NO2 medium was selected as the optimal condition to activate the siderophore BGC for the scale-up fermentation. Furthermore, these results demonstrated that culture regulation of the one strain–many compounds (OSMAC) strategy emerged as a simple and effective way to activate silent BGCs.

### 2.4. Molecular Networking Analysis of Siderophore Extracts

**Figure 5:**
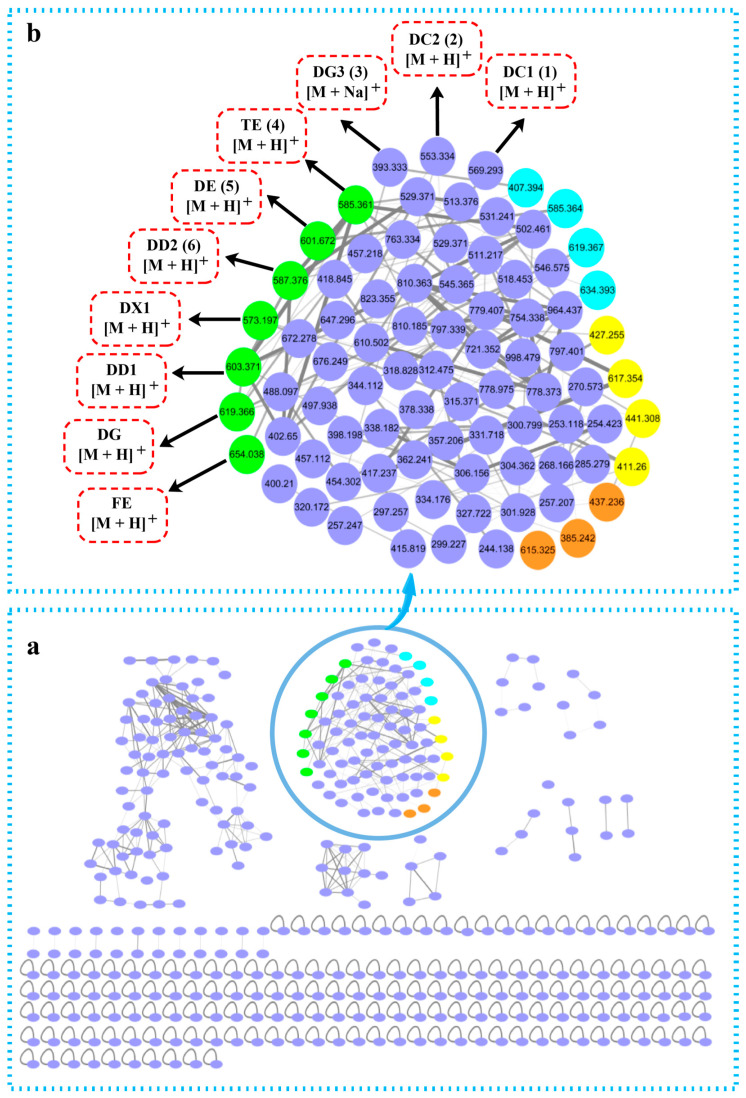
Molecular networking analysis of the siderophore extracts of *S. nanhaiensis* 12A09^T^ based on HPLC-MS/MS and GNPS. (**a**) The entire molecular networking. (**b**) The cluster corresponding to siderophore derivatives. DC1: desferrioxamine C1; DC2: desferrioxamine C2; DG3: desferrioxamine G3; TE: terragine E; DE: desferrioxamine E; DD2: desferrioxamine D2; DX1: desferrioxamine X1; DD1: desferrioxamine D1; DG: desferrioxamine G; FE: ferrioxamine E. The green, purple, yellow, orange, and blue nodes represent the recognized, uncharacterized, acetylation, methylation, and oxidation of desferrioxamine derivatives, respectively.

To further explore the siderophores produced by *S. nanhaiensis* 12A09^T^, the fermentation extracts were analyzed via the GNPS web platform. The molecular network revealed eight clusters (each containing at least three nodes) comprising 397 nodes in total, 14 clusters with two nodes each, and 168 unconnected singleton nodes (Figure 5a). Among them, the protonated molecular ion (*m*/*z*: 244.138–998.479) was identified as the primary component of the desferrioxamines (Figure 5b), determined by their consistent retention time in both HPLC-MS/MS and HPLC-DAD analyses.

The cluster mentioned above was further annotated as the “desferrioxamine molecular family” based on hits in the GNPS library. Notably, only seven nodes were identified (*m*/*z* 585.361, 601.672, 587.376, 573.197, 603.370, 619.366, and 654.038), which were recognized as terragine E (TE) (**4**), desferrioxamine E (DE) (**5**), desferrioxamine D2 (DD2) (**6**), desferrioxamine X1 (DX1), desferrioxamine D1 (DD1), desferrioxamine G (DG), and ferrioxamine E (FE), respectively (green nodes, Figure 5b and Table 3). The yellow nodes, orange nodes, and blue nodes may represent acetylation, methylation, and oxidation desferrioxamine derivatives (Figure 5b and Table 3). The identification of uncharacterized nodes (purple nodes) in the protonated molecular ion cluster suggested the presence of some potentially new desferrioxamine derivatives (Figure 5 and Table 3).

### 2.5. Structural Elucidation

**Figure 6:**
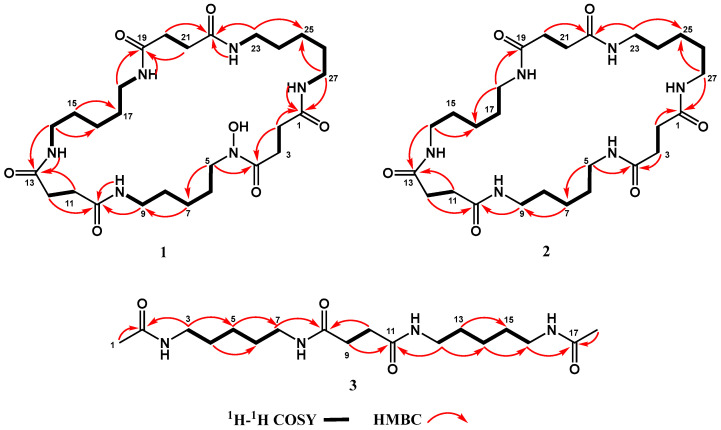
Key ^1^H-^1^H COSY and HMBC correlations of compounds **1**–**3**.

Desferrioxamine C1 (**1**) was isolated in the form of a white powder. The analysis of the HRESIMS data (*m*/*z* 591.3461 [M + Na]^+^) revealed the molecular formula C_27_H_48_N_6_O_7_, which accounts for seven degrees of unsaturation. An analysis of the 1D NMR spectra (Table 4 and Figures S2 and S3) and HSQC spectroscopic data (Figure S4) revealed the presence of six amide carbonyl groups and twenty-one methylene groups (six of which were nitrogenous). Although the 1D NMR spectra of **1** are very similar to those of desferrioxamine E (**5**) [23] (Table S2), the presence of disproportionate signals of methylene carbons indicated the loss of the characteristic 3-fold symmetry observed in desferrioxamine E. The integral of the ^1^H NMR and ^1^H-^1^H COSY correlations revealed the presence of three cadaverine (NCH_2_CH_2_CH_2_CH_2_CH_2_N) and three succinyl (COCH_2_CH_2_CO) moieties in **1** (Figure 6 and Figure S5). The chemical shifts of the terminal methylene protons for each cadaverine unit were observed at *δ*_H_ 3.45 and 2.98, with corresponding *δ*_C_ values of 46.8 and 38.3. The analysis of 1D NMR revealed that one cadaverine unit exhibited NOH (terminal methylene at *δ*_H_ 3.45 and *δ*_C_ 46.8) and NH (*δ*_H_ 2.98 and *δ*_C_ 38.3) at the two ends, while the other unit displayed NH at both ends, which was further supported by the molecular formula (desferrioxamine E was 32 Da larger than that of **1**). HMBC correlations of H_2_-2, H_2_-27/C-1 and H_2_-3, H_2_-5/C-4, along with ^1^H-^1^H COSY correlations of H_2_-2/H_2_-3 identified an unsymmetrical succinyl residue in **1**. HMBC correlations of H_2_-9, H_2_-12/C-10, H_2_-11, H_2_-14/C-13, H_2_-18/C-19, and H_2_-20, H_2_-23/C-22 showed that the two succinyl residues were linked to the two cadaverine moieties via amide bonds as in desferrioxamine E (Figure 6 and Figure S6). Hence, **1** was identified as a new desferrioxamine derivative and named desferrioxamine C1.

Desferrioxamine C2 (**2**) was isolated in the form of a white powder. The analysis of the HRESIMS data (*m*/*z* 575.3510 [M + Na]^+^) revealed the molecular formula C_27_H_48_N_6_O_6_, which accounts for seven degrees of unsaturation. The analysis of the 1D NMR spectra (Table 4 and Figures S7 and S8) and HSQC spectroscopic data (Figure S9) revealed that **2** possessed proportionate methylene carbon signals, indicating the retention of the characteristic 3-fold symmetry. The integral of the ^1^H NMR and ^1^H-^1^H COSY correlations indicated that **2** possessed three cadaverine (NCH_2_CH_2_CH_2_CH_2_CH_2_N) and three succinyl (COCH_2_CH_2_CO) moieties (Figure 6 and Figure S10). The analysis of 1D NMR of **2** showed that the cadaverine units had no NOH. The connections between the succinyl and cadaverine units were determined by HMBC via amide bonds as in desferrioxamine C1 (**1**) (Figure 6 and Figure S11). Hence, **2** was identified as a new desferrioxamine derivative and named desferrioxamine C2.

Desferrioxamine G3 (**3**) was isolated in the form of a white powder. The analysis of the HRESIMS data (*m*/*z* 393.2460 [M + Na]^+^) revealed the molecular formula C_18_H_34_N_4_O_4_, which accounts for four degrees of unsaturation. The analysis of the 1D NMR spectra (Table 4 and Figures S12 and S13) and HSQC spectroscopic data (Figure S14) revealed the presence of four amide carbonyl groups, twelve methylene groups (four of which were nitrogenous), and two methyl groups. The integral of the ^1^H NMR and ^1^H-^1^H COSY correlations revealed the presence of two cadaverine (NCH_2_CH_2_CH_2_CH_2_CH_2_N) and one succinyl (COCH_2_CH_2_CO) moieties in **3** (Figure 6 and Figure S15). HMBC correlations from H_3_-1 to C-2 and from H_3_-18 to C-17 identified two acetamide residues in **3**. The connections between the acetamide residues and the cadaverine units were determined by HMBC correlations from H_2_-3 to C-2 and from H_2_-16 to C-17. The connections between the succinyl and cadaverine units were via amide bonds as in desferrioxamine C1 (**1**), which were further determined by HMBC (Figure 6 and Figure S16). Hence, **3** was identified as a new desferrioxamine derivative and named desferrioxamine G3.

The remaining three known desferrioxamine derivatives were identified as terragine E (**4**) [24] (Table S3), desferrioxamine E (**5**) [23] (Table S4), and desferrioxamine D2 (**6**) [25] (Table S5) by a comparison of their NMR data with previously published references. In addition, although many potentially undescribed desferrioxamine derivatives could be envisioned by LC-MS/MS molecular networking analysis, a large part of them have not been isolated and characterized due to low yields.

### 2.6. Ferric Iron-Chelating Activity

In the CAS assay, **4** and **5** showed moderate Fe (III)-complexing capability (EC_50_ = 45.63 ± 7.40 and 26.30 ± 2.86 μM), weaker than the positive control desferrioxamine mesylate (DFOM) (EC_50_ = 11.68 ± 1.81 μM), and no chelating properties were observed for **1**–**3** and **6** (Figure S17). These results suggested that both the presence and quantity of the hydroxamate moiety played a critical role in mediating their interaction with iron [26]. In view of the fact that **6** displayed no chelating property, the characteristic 3-fold symmetry of desferrioxamine derivatives was essential for ferric iron-chelating activity. Compound **5** has been recognized as a detoxification agent for its superior Fe (III)-chelating capability [25].

### 2.7. Bioassay of Plant Growth Regulatory Activity

**Figure 7:**
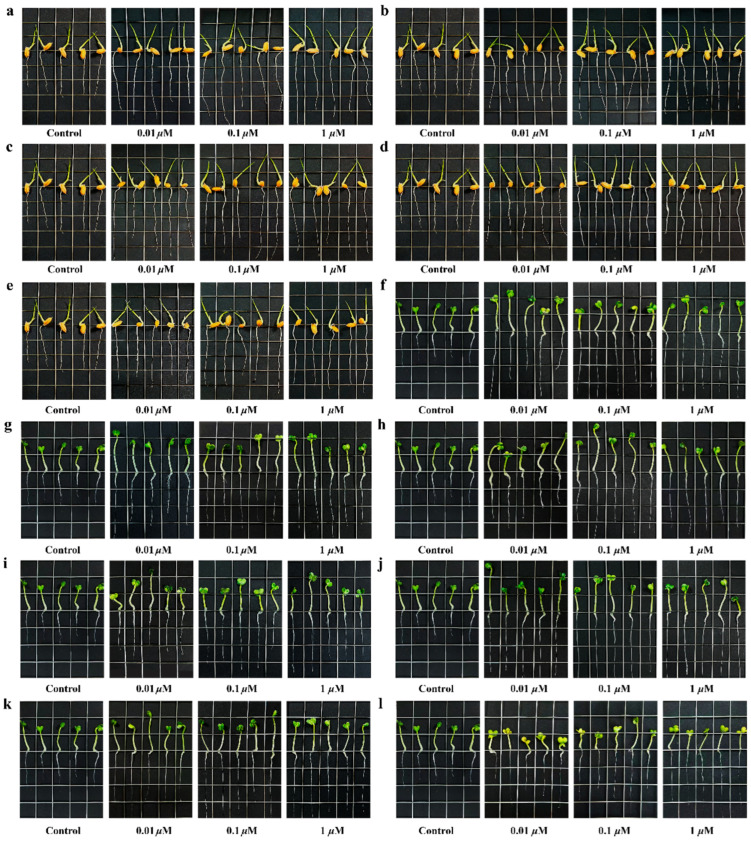
Physiological effects of compounds **1**, **4**–**6**, and GA (**a**–**e**) at various concentrations on root growth of *Oryza sativa*; physiological effects of compounds **1**–**6**, and GA (**f**–**l**) at various concentrations on root growth of *Brassica campestris*. The unit length of the boxes in each image (**a**–**l**) is 1 cm.

Compounds **1**–**6** and the positive control GA were assessed for their ability to regulate plant growth, focusing on the seedlings of *Oryza sativa* and *Brassica campestris*. As shown in Table 5 and Figure 7, compounds **1** and **4**–**6** showed growth-promoting effects on the seedlings of *Oryza sativa*, and compounds **1**–**6** showed growth-promoting effects on the seedlings of *Brassica campestris*. All active compounds exhibited a concentration-dependent relationship. Among them, compounds **1**, **5**, and **6** significantly boosted the root length in *Oryza sativa* seedlings, increasing it by 24%, 24%, and 18%, respectively, equivalent to the positive control GA (26%) at 1 μM (Table 5 and Figure 7). Compounds **1**, **5**, and **6** significantly boosted root length in *Brassica campestris* seedlings, increasing it by 136%, 136%, and 143%, respectively, equivalent to the positive control GA (143%) at 1 μM (Table 5 and Figure 7). However, compound **4** (12%) displayed weaker effects on the seedlings of *Oryza sativa* than GA (26%) at 1 μM, and compounds **2**–**4** (107%, 100%, and 79%) showed weaker effects on the seedlings of *Brassica campestris* than GA (143%) at 1 μM (Table 5 and Figure 7).

Roots, as the primary organ for nutrient absorption in plants, are increasingly valued for nourishing and strengthening roots in agricultural production. However, relatively few PGRs have been developed that specifically target root growth [4]. Compounds **1**, **5**, and **6** significantly increase root length, equivalent to, or even superior to, the positive control GA (Table 5 and Figure 7), suggesting excellent potential as PGRs targeting root growth. Among them, compound **5** exhibits both root growth-promoting and excellent ferric iron-chelating activities, making it a particularly competitive dual-functional PGR and siderophore. This is the first report demonstrating the plant growth-promoting activity of siderophore secondary metabolites. Notably, marine natural products as PGRs have been scarcely reported to date (only alginate oligosaccharide) [9]. Therefore, compounds **1**, **5**, and **6** isolated from marine-derived *S. nanhaiensis* 12A09^T^ provide an example for the application of marine natural products as important resources in agriculture.

According to the above data, it was found that the plant growth-promoting and the ferric iron-chelating activities of compounds **1**–**6** exhibited no direct correlation, which suggested that the potential mechanism by which these desferrioxamines promote root development in plants may not rely on iron transport. However, the mechanism requires further experimental verification.

## 3. Materials and Methods

### 3.1. General Experimental Procedures

UV spectra were measured by a UNICO 2365 spectrophotometer (Unico, Shanghai, China). IR data were obtained using a Thermo Fisher Nicolet 6700 FT-IR spectrometer (Thermo Fisher Scientific, Waltham, MA, USA). A Bruker-AV-600 NMR spectrometer (Bruker, Karlsruhe, Germany) was used to obtain all NMR spectra. A Dionex UltiMate 3000 system (Thermo Fisher Scientific, Waltham, MA, USA) was used to carry out semipreparative reversed-phase HPLC separation. HRESIMS data were analyzed by a Thermo Scientific Q Exactive mass spectrometer (Thermo Fisher Scientific, Waltham, MA, USA).

### 3.2. Actinomycete Strain

*S. nanhaiensis* 12A09^T^ was isolated from the marine sediment samples obtained from the South China Sea, and it has been deposited at the China Center for Type Culture Collection with the number CCTCC AB 2013140. This strain was identified as a novel species based on phylogenetic analysis of 16S rRNA gene sequences, genotypic, and phenotypic data [27].

### 3.3. Genome Sequencing and BGCs Mining

The genome of *S. nanhaiensis* 12A09^T^ was sequenced by Shanghai Personalbio Technology Co., Ltd. (Shanghai, China) with a combination of next-generation sequencing using Illumina MiSeq and long-read sequencing using PacBio RS platforms. Then, bioinformatics analysis succeeded in assembling the whole genome of *S. nanhaiensis* 12A09^T^ from the generated sequence data. AntiSMASH 8.0 [28], 2ndFind, and BLAST+2.17.0 were used for the analysis of the potential BGCs of *S. nanhaiensis* 12A09^T^, especially the siderophore BGC, followed by manual verification.

### 3.4. Culture Regulation for Activating the Siderophore BGC

According to the OSMAC strategy, some media were adopted to active silenced siderophore BGC, including NO2 medium (2% glucose, 1% soluble starch, 1% yeast extract, 1% tryptone, 0.3% beef extract, 0.2% CaCO_3_, 0.05% K_2_HPO_4_, 0.05% MgSO_4_, and 3% artificial sea salt, pH = 7.0), NM2 medium (2% glycerin, 1% lactose, 0.5% soya peptone, 0.3% yeast extract, 0.15% NH_4_NO_3_, 0.1% glucose, 0.02% trace elements), ISP3 medium (2% oat kernel flour and 0.1% trace elements, pH 7.0), and PSB medium (20% boiled and mashed potatoes and 2% sucrose, pH 7.0). The strain *S. nanhaiensis* 12A09^T^ was cultured at 180 rpm at 28 °C in 250 mL Erlenmeyer flasks for 7 days. The mycelia and supernatant were obtained by centrifuging (8000 rpm for 30 min). The supernatant was extracted by HP20 macroporous adsorption resin to obtain crude extracts.

### 3.5. CAS Plate Assay

The production of siderophores from the above crude extracts was tested by a CAS plate assay [29]. CAS medium: 72.9 mg hexadecyltrimethylammonium bromide (CTAB), 60.5 mg CAS, 10 mL Fe^3+^ solution (10 mM HCl and 1 mM FeCl_3_·6H_2_O), 2 g agar, and 100 mL 0.1 M PBS (pH 6.8). The CAS medium was sterilized for 30 min at 121 °C and diluted tenfold before use. The above crude extracts (10 mg/mL) were added to diluted CAS medium to observe color changes after incubation at 37 °C for 48 h.

### 3.6. Fermentation and Extraction

The strain was cultured using NO2 medium and maintained at 28 °C for 7 days at 180 rpm. The mycelia and supernatant were separated by centrifuging (4000 rpm for 20 min). The supernatant (50 L) was extracted using HP20 macroporous adsorption resin and subsequently filtered. The resin was then eluted three times using methanol, which was evaporated to obtain a crude extract weighing 43.1 g.

### 3.7. LC-MS/MS Molecular Networking Analysis

The crude extract with iron-chelating activity was analyzed by LC-MS/MS with an HPLC system coupled to a Q-Exactive mass spectrometer (Thermo Fisher Scientific, MA, USA). The mobile phase consisted of A (0.05% formic acid in H_2_O) and B (CH_3_OH). The compounds were eluted via a gradient program from 10% to 100% B over 30 min, then held for 5 min with 100% B, and returned to 10% B at 35 °C at 1.0 mL/min. ESI conditions were set with a sheath gas flow rate of 40 arb, a source voltage of 3.8 kV, and a capillary temperature of 320 °C. The ESI acquisition was performed in data-dependent scan mode with a scan range of m/z 150-1500. Subsequently, the raw data were converted into mzXML format by MSConvert (ProteoWizard 3.0.20083 64-bit) [30]. A molecular network was constructed using the GNPS data analysis workflow [31]. Cytoscape 3.6.1 software was used to visualize the molecular networks [32]. The nodes represented parent *m*/*z* and edge thickness corresponded to cosine scores.

### 3.8. HPLC-DAD and Bioactivity Guided Isolation and Purification

The crude extract (43.1 g) was fractionated into seven fractions (A–G) over silica gel column chromatography with a CH_2_Cl_2_-MeOH gradient system from 100:0 to 0:100 (*v*/*v*). Then, the CAS assay of fractions A-G was tested. To isolate and purify the target fraction B (5.0 g), Sephadex LH-20 was employed, obtaining two subfractions B_1-2_. Fraction B_1_ was purified through semipreparative HPLC with a YMC-Pack-ODS-A column (250 × 10 mm, 5 µm) by a mobile phase consisting of methanol/water (45:55, *v*/*v*). This process yielded **1** (*t*_R_ = 15.0 min, 12.7 mg), **4** (*t*_R_ = 20.1 min, 16.5 mg), and **5** (*t*_R_ = 26.5 min, 9.7 mg). Fraction B_2_ was purified using semipreparative HPLC with a mobile phase of methanol/water (45:55, *v*/*v*) to gain **6** (*t*_R_ = 18.3 min, 1.9 mg). To isolate and purify the target fraction C (4.5 g), Sephadex LH-20 was employed to obtain a subfraction C, which was further separated by semipreparative HPLC with a mobile phase of methanol/water (30:70, *v*/*v*) to give **2** (*t*_R_ = 10.1 min, 11.5 mg) and **3** (*t*_R_ = 12.1 min, 4.6 mg).

Desferrioxamine C1 (**1**): white powder, UV (MeOH) *λ*_max_ (log *ε*) 229 (2.21) nm (Figure S18); IR (KBr) *ν*_max_ 3705, 3617, 3292, 2972, 2944, 2868, 1641, 1550, 1056, and 1022 cm^−1^ (Figure S19); (+)-HRESIMS *m*/*z* 591.3461 [M + Na]^+^ (calcd for C_27_H_48_N_6_O_7_Na^+^, 591.3477) (Figure S20).

Desferrioxamine C2 (**2**): white powder, UV (MeOH) *λ*_max_ (log *ε*) 221 (1.99) nm (Figure S21); IR (KBr) *ν*_max_ 3705, 3617, 3290, 2972, 2929, 2867, 1636, 1559, 1056, 1022, and 1010 cm^−1^ (Figure S22); (+)-HRESIMS *m*/*z* 575.3510 [M + Na]^+^ (calcd for C_27_H_48_N_6_O_6_Na^+^, 575.3527) (Figure S23).

Desferrioxamine G3 (**3**): white powder, UV (MeOH) *λ*_max_ (log *ε*) 210 (1.21) nm (Figure S24); IR (KBr) *ν*_max_ 3705, 3617, 3292, 2972, 2945, 2868, 1642, 1547, 1056, 1022, and 1010 cm^−1^ (Figure S25); (+)-HRESIMS *m*/*z* 393.2460 [M + Na]^+^ (calcd for C_18_H_34_N_4_O_4_Na^+^, 393.2472) (Figure S26).

### 3.9. Ferric Iron-Chelating Activity

To evaluate the iron-chelating ability of compounds **1**–**6**, a modified CAS was conducted [11]. CTAB (72.9 mg) was diluted in 40 mL of H_2_O at 35 °C. An amount of 50 mL of 2 mM aqueous CAS mixed with 10 mL of iron solution (10 mM HCl and 1 mM FeCl_3_·6H_2_O) was added to the CTAB solution to obtain the CTAB-CAS-Fe (III) solution. Compounds **1**–**6** were dissolved in DMSO and then added to sterile water to obtain final concentrations of 5 μM, 10 μM, 50 μM, 100 μM, and 200 μM. A total of 100 μL of these solutions was loaded in a 96-well plate. Then, 100 μL of blank controls (the same amount of water) or compounds at different concentrations were added to each well. The color changed from blue to orange or red after incubation for 3 h at 37 °C. UV–vis absorbance was measured at 630 nm by a plate reader. DFOM served as a positive control. Absorbance of CAS solutions at 630 nm was reduced by 50% to determine the EC_50_ values. The calculation of the EC_50_ values was performed through GraphPad Prism 9.2.

### 3.10. Bioassay of Plant Growth Regulatory Activity

The growth-promoting activities of compounds **1**–**6** were evaluated using seedlings of *Oryza sativa* and *Brassica campestris* according to a slightly modified method previously described [33]. Briefly, these seeds were surface-sterilized with 75% ethanol for 5 min, and then they were washed five times with sterile water. Following sterilization, the seeds were pregerminated at 25 °C for 1 or 2 days in sterile water. Compounds **1**–**6** were dissolved in DMSO and then added to sterile water to obtain final concentrations of 0.01 μM, 0.1 μM, and 1 μM. GA was selected as a positive control. Finally, the seedlings were grown under a 16 h light/8 h dark cycle without soil, and the root length (*n* = 10) was measured using a ruler with the smallest scale in mm after 2–4 days.

## 4. Conclusions

In conclusion, based on the SPLSD strategy, a unique deferoxamine-like BGC of the new species *S. nanhaiensis* 12A09^T^ was identified and activated. Three new desferrioxamine derivatives (**1**–**3**) and three known ones (**4**–**6**) were isolated from *S. nanhaiensis* 12A09^T^. In a ferric iron-chelating assay, **4** and **5** showed moderate Fe (III)-complexing capability. In a plant-growth regulatory assay, **1**, **5**, and **6** significantly boosted the root length in *Oryza sativa* and *Brassica campestris* seedlings, equivalent to GA. Thus, these findings provide direct evidence for the first time for the growth-promoting activity of siderophores. Meanwhile, compound **5** exhibited both root growth-promoting and excellent ferric iron-chelating activities, which could be used as a siderophore PGR lead compound.

## Figures and Tables

**Table 1:** Biosynthetic gene clusters identified in the *S. nanhaiensis* 12A09^T^ genome via antiSMASH 8.0.

Clusters	Type	From	To	Biosynthetic Potential
Cluster 1	Lassopeptide	191,959	214,488	Streptomonomicin
Cluster 2	T2PKS	605,576	653,305	Unknown
Cluster 3	Terpene	1,381,481	1,407,212	Isorenieratene
Cluster 4	Guanidinotides	1,441,572	1,464,125	Unknown
Cluster 5	Butyrolactone	1,583,381	1,594,478	Unknown
Cluster 6	Siderophore	1,710,310	1,740,625	Desferrioxamine-like
Cluster 7	Terpene-precursor	1,948,404	1,969,495	Unknown
Cluster 8	Butyrolactone	2,314,592	2,325,629	Unknown
Cluster 9	T3PKS	2,485,075	2,526,133	Unknown
Cluster 10	Terpene	2,566,315	2,587,691	Unknown
Cluster 11	RiPP-like	2,592,565	2,602,861	Unknown
Cluster 12	NRPS	3,068,416	3,150,338	Omnipeptin-like
Cluster 13	Terpene-precursor	3.217,990	3,240,236	Unknown
Cluster 14	T1PKS	3,809,298	3,855,021	Unknown
Cluster 15	NRPS	4,127,061	4,178,721	Coelibactin
Cluster 16	Phosphonate	4,383,958	4,394,830	Unknown
Cluster 17	NRPS-like	4,411,046	4,454,201	Guanipiperazine A/B-like
Cluster 18	Terpene	5,071,300	5,092,994	Unknown
Cluster 19	T1PKS	5,706,685	5,764,401	Unknown
Cluster 20	Ectoine	6,320,017	6,330,418	Ectoine
Cluster 21	T2PKS	6,626,639	6,699,116	Unknown
Cluster 22	Lanthipeptide-class-iv	6,878,044	6,900,620	Duramycin
Cluster 23	PKS-like	7,053,968	7,096,753	Unknown

**Table 2:** Functional annotation of the desferrioxamin-like BGC in *S. nanhaiensis* 12A09^T^.

Protein	Size (aa)	Proposed Function	Accession	Identities	Positives	Homologous Protein
ORF-1	577	Major facilitator superfamily transporter	XKK40617.1	99%	99%	
ORF-2	186	TetR family transcriptional regulator	GAA1444975.1	98%	98%	
ORF1	547	*L*-2, 4-diaminobutyrate decarboxylase	SFL00654.1	67%	75%	DesA
ORF2	482	Lysine/ornithine *N*-monooxygenase	XKK40621.1	99%	99%	DesB
ORF3	206	*N*-acetyltransferase	SIO88002.1	69%	76%	DesC
ORF4	644	Siderophore biosynthesis protein	MEU1626482.1	71%	81%	DesD
ORF5	267	Methyltransferase	XKK42002.1	94%	94%	
ORF6	538	Acyl-CoA synthetase	SHJ64988.1	80%	85%	
ORF7	547	Acyl-CoA transferase	SIO85325.1	73%	77%	

**Table 3:** Identified nodes in desferrioxamine molecular clusters based on GNPS.

Observed Mass Peak (*m*/*z*)	Molecular Formula	Assignment	Post-Modification
393.333 [M + Na]^+^	C_18_H_34_N_4_O_4_	Desferrioxamine G3 (**3**)	
553.334 [M + H]^+^	C_27_H_48_N_6_O_6_	Desferrioxamine C2 (**2**)	
569.293 [M + H]^+^	C_27_H_48_N_6_O_7_	Desferrioxamine C1 (**1**)	
573.197 [M + H]^+^	C_25_H_44_N_6_O_9_	Desferrioxamine X1	
585.361 [M + H]^+^	C_27_H_48_N_6_O_8_	Terragine E (**4**)	
587.376 [M + H]^+^	C_26_H_46_N_6_O_9_	Deferrioxamine D2 (**6**)	
601.672 [M + H]^+^	C_27_H_48_N_6_O_9_	Desferrioxamine E (**5**)	
603.370 [M + H]^+^	C_27_H_50_N_6_O_9_	Desferrioxamine D1	
619.366 [M + H]^+^	C_27_H_50_N_6_O_10_	Desferrioxamine G	
654.038 [M + H]^+^	C_27_H_45_FeN_6_O_9_	Ferrioxamine E	
411.260 [M − H]^−^	C_20_H_36_N_4_O_5_	Desferrioxamine G3 (**3**) + acetyl	Acetylation
427.255 [M − H]^−^	C_19_H_32_N_4_O_7_	Avaroferrin + acetyl	Acetylation
441.308 [M − H]^−^	C_20_H_34_N_4_O_7_	Bisucaberin + acetyl	Acetylation
617.354 [M + Na]^+^	C_29_H_50_N_6_O_7_	Desferrioxamine C2 (**2**) + acetyl	Acetylation
385.242 [M + H]^+^	C_19_H_36_N_4_O_4_	Desferrioxamine G3 (**3**) + CH_3_	Methylation
437.236 [M + Na]^+^	C_19_H_34_N_4_O_6_	Bisucaberin + CH_3_	Methylation
615.325 [M + H]^+^	C_28_H_50_N_6_O_9_	Desferrioxamine E (**5**) + CH_3_	Methylation
407.394 [M + Na]^+^	C_18_H_32_N_4_O_4_	Desferrioxamine G3 (**3**) − 2H	Oxidation
585.364 [M + H]^+^	C_27_H_48_N_6_O_8_	Desferrioxamine C1 (**1**) + O	Oxidation
619.367 [M + H]^+^	C_27_H_50_N_6_O_10_	Desferrioxamine D1 + O	Oxidation
634.393 [M + NH_4_]^+^	C_27_H_48_N_6_O_10_	Desferrioxamine E (**5**) + O	Oxidation

**Table 4:** ^1^H and ^13^C NMR data for **1**–**3** (600/150 MHz, *δ* in ppm).

Position	1 ^*^		2 ^#^		3 ^#^	
	*δ*_H_ (*J* in Hz)	*δ*_C_, Type	*δ*_H_ (*J* in Hz)	*δ*_C_, Type	*δ*_H_ (*J* in Hz)	*δ*_C_, Type
1		171.1 C		174.6 C	1.92 s ^a^	22.5 CH_3_
2	2.26 m ^a^	29.9 CH_2_	2.47 s ^a^	32.5 CH_2_		173.2 C
3	2.58 m	27.5 CH_2_	2.47 s ^a^	32.5 CH_2_	3.15 m ^b^	40.3 CH_2_
4		172.1 C		174.6 C	1.51 m ^c^	30.0 CH_2_
5	3.45 m	46.8 CH_2_	3.17 t (6.7) ^b^	40.1 CH_2_	1.35 m ^d^	25.2 CH_2_
6	1.48 m	25.8 CH_2_	1.51 m ^c^	29.9 CH_2_	1.51 m ^c^	30.0 CH_2_
7	1.20 m ^b^	23.1 CH_2_	1.35 m ^d^	25.0 CH_2_	3.15 m ^b^	40.3 CH_2_
8	1.34 m ^c^	28.7 CH_2_	1.51 m ^c^	29.9 CH_2_		174.5 C
9	2.98 m ^d^	38.2 CH_2_	3.17 t (6.7) ^b^	40.1 CH_2_	2.45 s ^e^	32.3 CH_2_
10		171.2 C		174.6 C	2.45 s ^e^	32.3 CH_2_
11	2.26 m ^a^	31.0 CH_2_	2.47 s ^a^	32.5 CH_2_		174.5 C
12	2.26 m ^a^	31.0 CH_2_	2.47 s ^a^	32.5 CH_2_	3.15 m ^b^	40.3 CH_2_
13		171.4 C		174.6 C	1.51 m ^c^	30.0 CH_2_
14	2.98 m ^d^	38.3 CH_2_	3.17 t (6.7) ^b^	40.1 CH_2_	1.35 m ^d^	25.2 CH_2_
15	1.34 m ^c^	28.7 CH_2_	1.51 m ^c^	29.9 CH_2_	1.51 m ^c^	30.0 CH_2_
16	1.20 m ^b^	23.5 CH_2_	1.35 m ^d^	25.0 CH_2_	3.15 m ^b^	40.3 CH_2_
17	1.34 m ^c^	28.7 CH_2_	1.51 m ^c^	29.9 CH_2_		173.2 C
18	2.98 m ^d^	38.2 CH_2_	3.17 t (6.7) ^b^	40.1 CH_2_	1.92 s ^a^	22.5 CH_3_
19		171.3 C		174.6 C		
20	2.26 m ^a^	31.0 CH_2_	2.47 s ^a^	32.5 CH_2_		
21	2.26 m ^a^	31.0 CH_2_	2.47 s ^a^	32.5 CH_2_		
22		171.3 C		174.6 C		
23	2.98 m ^d^	38.3 CH_2_	3.17 t (6.7) ^b^	40.1 CH_2_		
24	1.34 m ^c^	28.7 CH_2_	1.51 m ^c^	29.9 CH_2_		
25	1.20 m ^b^	23.5 CH_2_	1.35 m ^d^	25.0 CH_2_		
26	1.34 m ^c^	28.7 CH_2_	1.51 m ^c^	29.9 CH_2_		
27	2.98 m ^d^	38.2 CH_2_	3.17 t (6.7) ^b^	40.1 CH_2_		
-NH	7.72 s					
N-OH	9.58 s					

* Measured in DMSO-*d*_6_. ^#^ Measured in CD_3_OD-*d*_4_. Superscripts a–e indicate overlapping.

**Table 5:** Root length (cm) of *Oryza sativa* and *Brassica campestris* treated with compounds **1**–**6** and gibberellin.

Compound	*Oryza sativa*			*Brassica campestris*		
	1 μM	0.1 μM	0.01 μM	1 μM	0.1 μM	0.01 μM
**1**	4.2 ± 0.2 ^***^	4.2 ± 0.2 ^***^	3.8 ± 0.4 ^**^	3.3 ± 0.2 ^***^	2.9 ± 0.3 ^***^	2.5 ± 0.2 ^***^
**2**	3.6 ± 0.3	2.8 ± 0.4	3.5 ± 0.3	2.9 ± 0.1 ^***^	2.7 ± 0.5 ^***^	2.7 ± 0.3 ^***^
**3**	3.4 ± 0.4	3.5 ± 0.3	3.0 ± 0.4	2.8 ± 0.3 ^***^	2.8 ± 0.3 ^***^	2.2 ± 0.3 ^***^
**4**	3.8 ± 0.3 ^**^	3.6 ± 0.2 ^*^	3.5 ± 0.2	2.5 ± 0.3 ^***^	2.4 ± 0.3 ^***^	2.1 ± 0.2 ^***^
**5**	4.2 ± 0.3 ^***^	4.2 ± 0.3 ^***^	4.1 ± 0.2 ^***^	3.3 ± 0.3 ^***^	3.0 ± 0.4 ^***^	2.9 ± 0.4 ^***^
**6**	4.0 ± 0.3 ^***^	3.9 ± 0.3 ^***^	3.5 ± 0.2	3.4 ± 0.3 ^***^	2.8 ± 0.4 ^***^	2.7 ± 0.4 ^***^
Gibberellin	4.3 ± 0.2 ^***^	4.2 ± 0.3 ^***^	4.1 ± 0.3 ^***^	3.4 ± 0.3 ^***^	3.0 ± 0.5 ^***^	2.8 ± 0.2 ^***^
Blank control		3.4 ± 0.2			1.4 ± 0.1	

*** *p* < 0.001, ** *p* < 0.01, * *p* < 0.05 when compared with the blank control.

## Data Availability

The authors confirm that the data supporting the findings of this study are available within the article and its Supplementary Materials.

## Supplementary Materials

The following supporting information can be downloaded at https://www.mdpi.com/article/10.3390/md24010007/s1, Figure S1: The systematic pipeline for efficient lead structure discovery from microbial natural products by promising strain selection and multi-omics mining (SPLSD); Figures S2–S16: 1D and 2D NMR spectra of compounds **1**–**3**; Figure S17: CAS assay results of compounds **1**–**6** and DFOM; Figures S18–S26: UV, IR, and HR-ESI-MS spectra of compounds **1**–**3**; Table S1: Number of genes associated with the 19 general COG functional categories; Table S2: ^1^H and ^13^C NMR data for **1** and **5** in DMSO-*d*_6_ (600/150 MHz, *δ* in ppm); Table S3: ^1^H and ^13^C NMR Data for **4** and terragine E in DMSO-*d*_6_ (600/150 MHz, *δ* in ppm); Table S4: ^1^H and ^13^C NMR Data for **5** and desferrioxamine E in DMSO-*d*_6_ (600/150 MHz, *δ* in ppm); Table S5: ^1^H and ^13^C NMR Data for **6** and desferrioxamine D2.
