# Supplementary material for: Genome Mining and Molecular Networking-Targeted Discovery of Siderophores with Plant Growth-Promoting Activities from the Marine-Derived Streptomonospora nanhaiensis 12A09T
Source: Mar Drugs. 2025 Dec 22;24(1):7. doi: 10.3390/md24010007 (PMC12842786; doi:10.3390/md24010007)
Supplement: Supplementary file 1 [file marinedrugs-24-00007-s001.zip › marinedrugs-4003088-supplementary.pdf]

Supporting Information

Genome Mining and Molecular Networking Targeted Discovery of Siderophores with Plant Growth-promoting Activities from the marine-derived *Streptomonospora nanhaiensis* 12A09^{T}

Yan Bai[1, †], Weixian Gao[1, †], Wendian Zhao[1], Amr A Arishi[2], Zhuo Shang[3], Jiangchun Hu[1], Huaqi Pan[1, *]

[1]CAS Key Laboratory of Forest Ecology and Silviculture, Institute of Applied Ecology, Chinese Academy of Sciences, Shenyang 110016, China

[2]School of Molecular Sciences, The University of Western Australia, 6009 Perth, WA, Australia

[3]School of Pharmaceutical Science, Shandong University, Jinan 250012, China

[*] Correspondence: panhq@iae.ac.cn; Tel.: +86-24-83970386

[†] These authors contributed equally to this work.

List of Supporting Information

Figure S1. The systematic pipeline for efficient lead structure discovery from microbial natural products by promising strain selection and multi-omics mining (SPLSD).

Figure S2. ^{1}H NMR (600 MHz, DMSO-d_6) spectrum of **1**.

Figure S3. ^{13}C NMR (150 MHz, DMSO-d_6) spectrum of **1**.

Figure S4. HSQC spectrum of **1**.

Figure S5. ^{1}H-^{1}H COSY spectrum of **1**.

Figure S6. HMBC spectrum of **1**.

Figure S7. ^{1}H NMR (600 MHz, CD_3OD-d_4) spectrum of **2**.

Figure S8. ^{13}C NMR (150 MHz, CD_3OD-d_4) spectrum of **2**.

Figure S9. HSQC spectrum of **2**.

Figure S10. ^{1}H-^{1}H COSY spectrum of **2**.

Figure S11. HMBC spectrum of **2**.

Figure S12. ^{1}H NMR (600 MHz, CD_3OD-d_4) spectrum of **3**.

Figure S13. ^{13}C NMR (150 MHz, CD_3OD-d_4) spectrum of **3**.

Figure S14. HSQC spectrum of **3**.

Figure S15. ^{1}H-^{1}H COSY spectrum of **3**.

Figure S16. HMBC spectrum of **3**.

Figure S17. CAS assay results of **1-6** and DFOM. Concentration-dependent CAS liquid well-plate assay and EC_{50} values.

Figure S18. UV spectrum of **1**.

Figure S19. IR spectrum of **1**.

Figure S20. (+)-HRESIMS spectrum of **1**.

Figure S21. UV spectrum of **2**.

Figure S22. IR spectrum of **2**.

Figure S23. (+)-HRESIMS spectrum of **2**.

Figure S24. UV spectrum of **3**.

Figure S25. IR spectrum of **3**.

Figure S26. (+)-HRESIMS spectrum of **3**.

Table S1 Number of genes associated with the 19 general COG functional categories.

Table S2 ^{1}H and ^{13}C NMR Data for **1** and **5** in DMSO-d_6 (600/150 MHz, δ in ppm).

Table S3 ^{1}H and ^{13}C NMR Data for **4** and terragine E in DMSO-d_6 (600/150 MHz, δ in ppm).

Table S4 ^{1}H and ^{13}C NMR Data for **5** and desferrioxamine E in DMSO-d_6 (600/150 MHz, δ in ppm).

Table S5 ^{1}H and ^{13}C NMR Data for **6** and desferrioxamine D2.

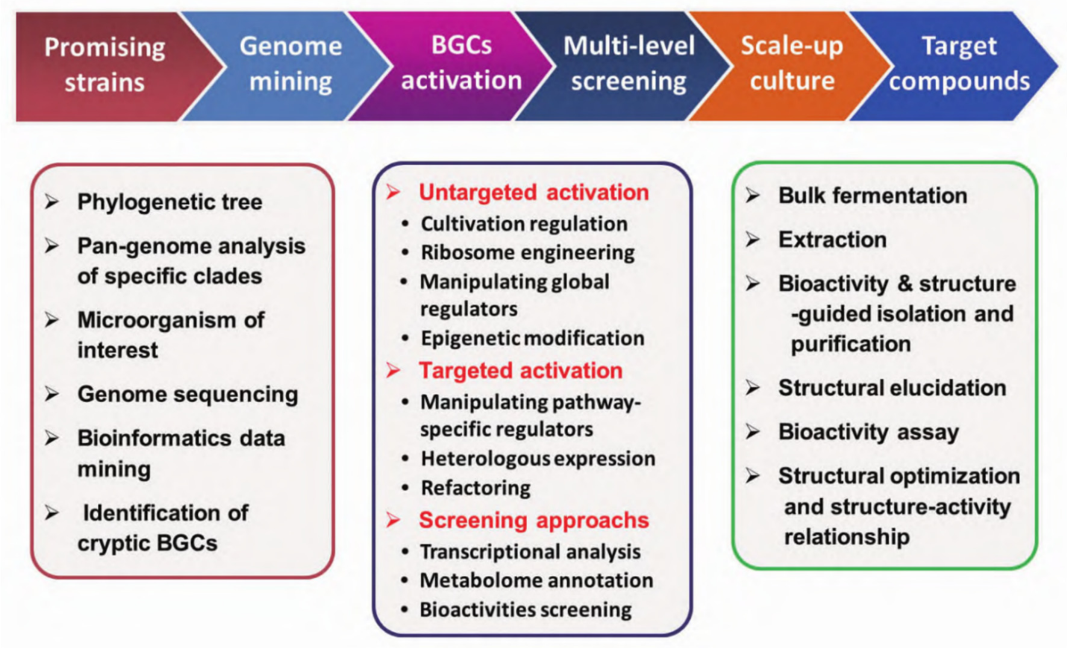

Figure S1. The systematic pipeline for efficient lead structure discovery from microbial natural products by promising strain selection and multi-omics mining (SPLSD) [1] (Cited from the literature: Pan, H.Q. Design and prospects of an efficient mining pipeline for microbial natural products in the post-genome era. Journal of Microbiology. (Chinese), 2022, 42 (3), 1−14. http://wswx.cnjournals.com/ch/reader/view_abstract.aspx?file_no=20220301&flag=1).

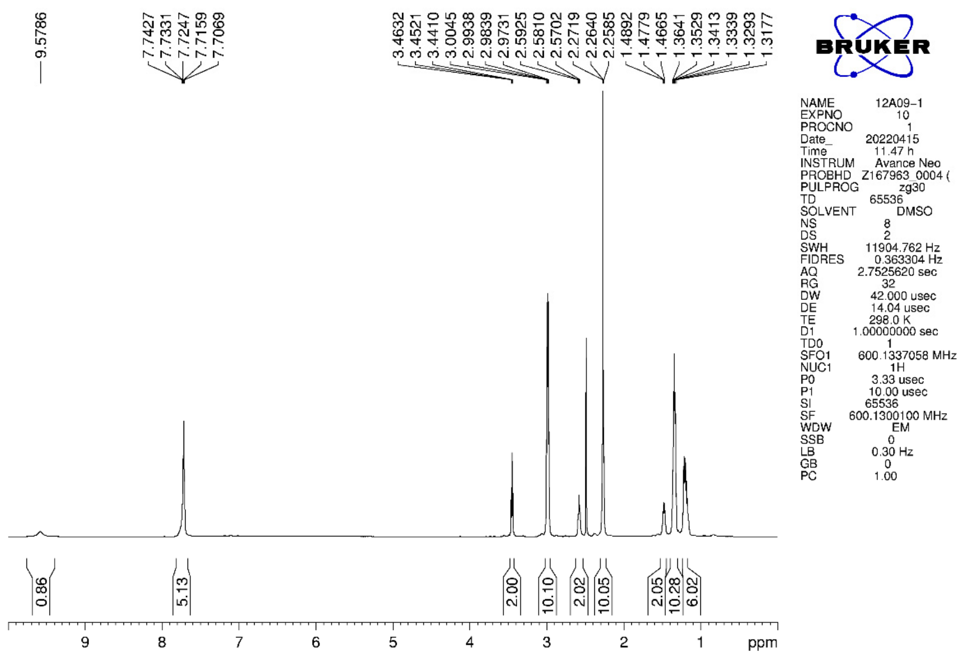

Figure S2. ^{1}H NMR (600 MHz, DMSO-d_6) spectrum of **1**

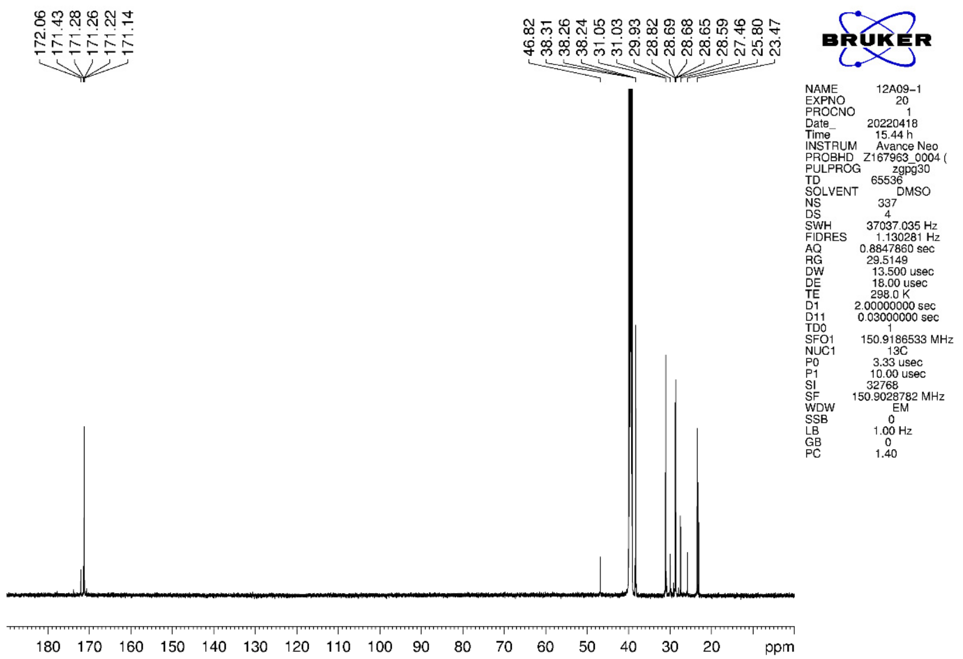

Figure S3. ^{13}C NMR (150 MHz, DMSO-d_6) spectrum of **1**

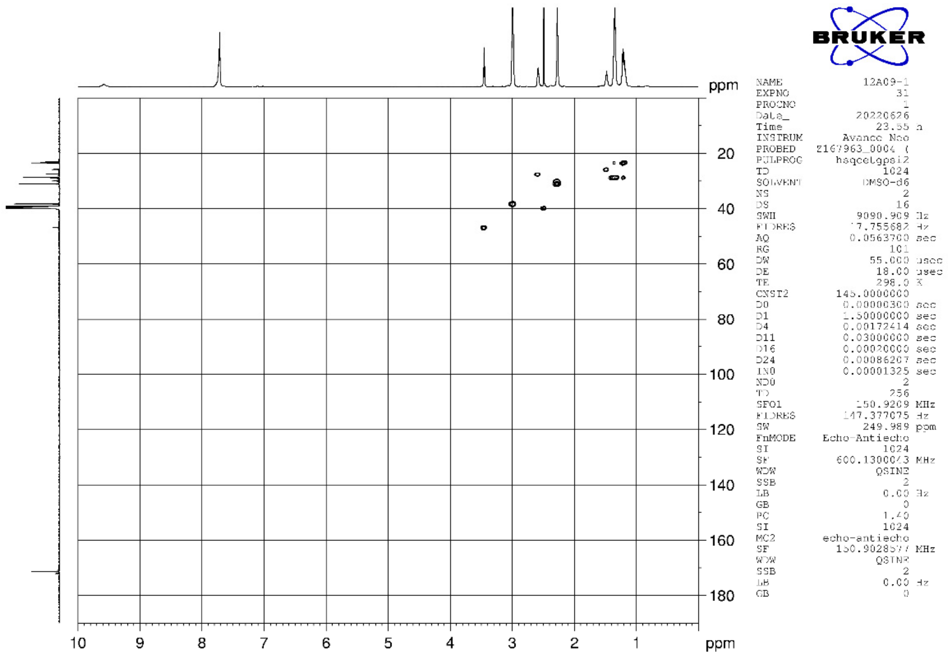

Figure S4. HSQC spectrum of **1**

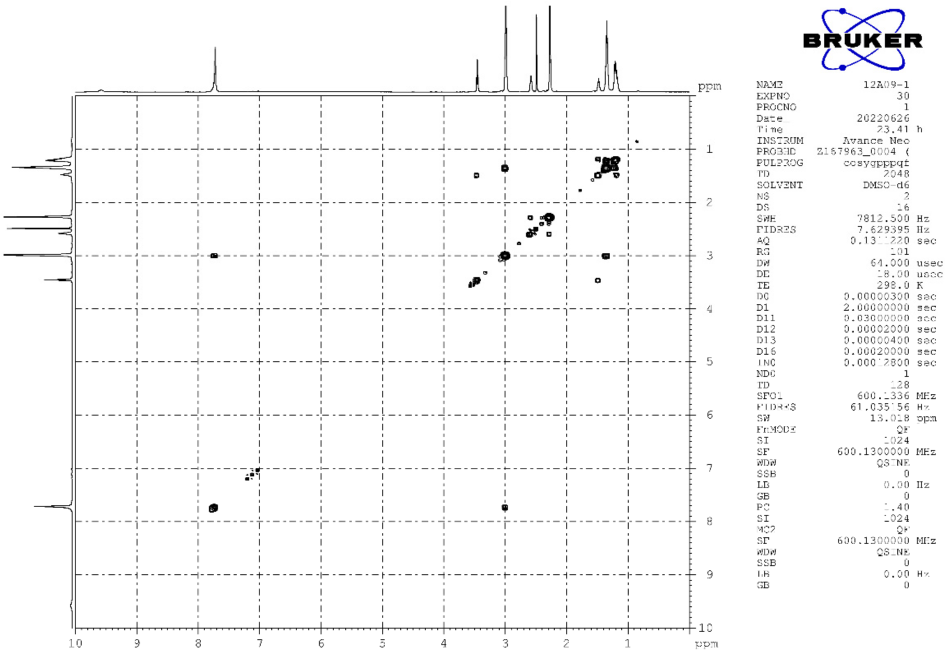

Figure S5. ^{1}H-^{1}H COSY spectrum of **1**

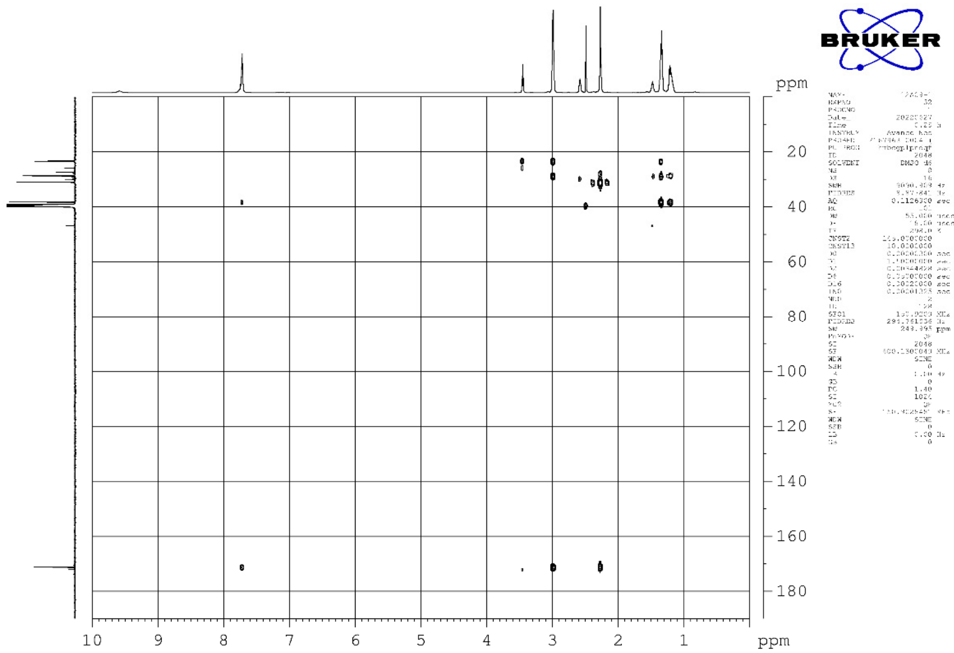

Figure S6. HMBC spectrum of **1**

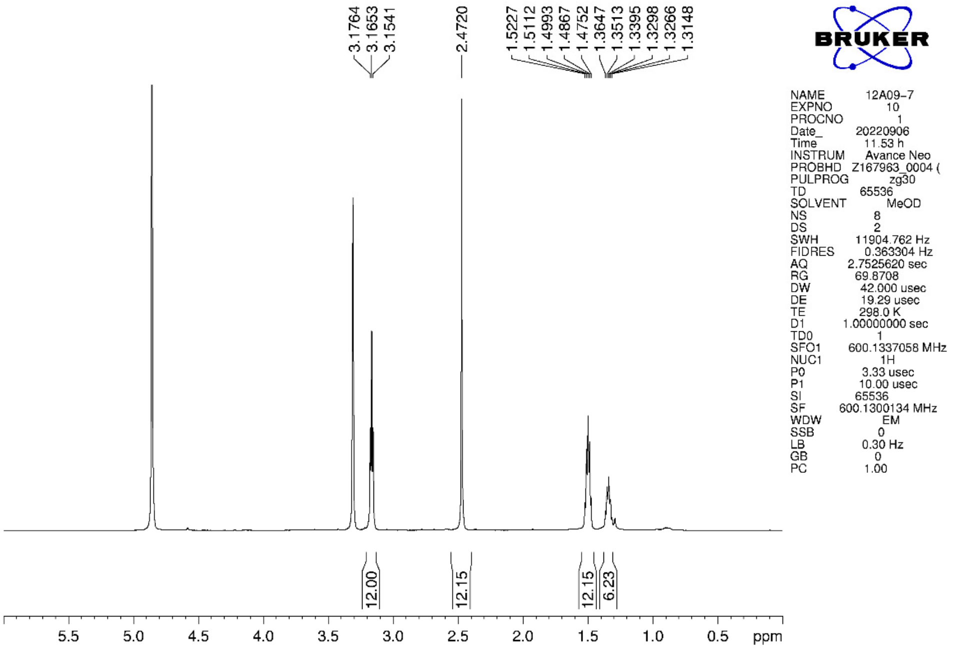

Figure S7. ^{1}H NMR (600 MHz, CD_3OD-d_4) spectrum of **2**

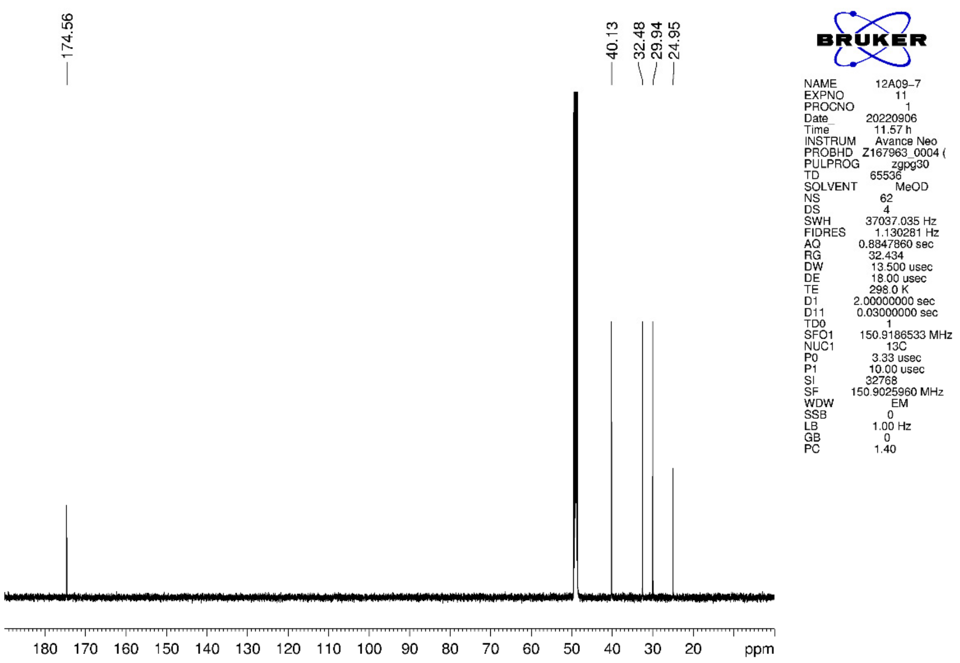

Figure S8. ^{13}C NMR (150 MHz, CD_3OD-d_4) spectrum of **2**

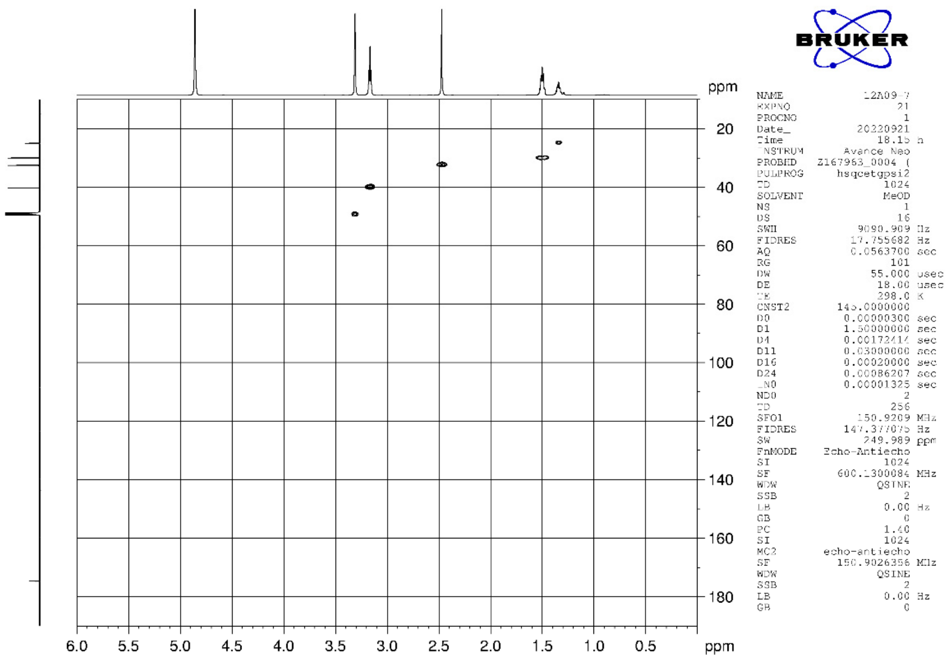

Figure S9. HSQC spectrum of **2**

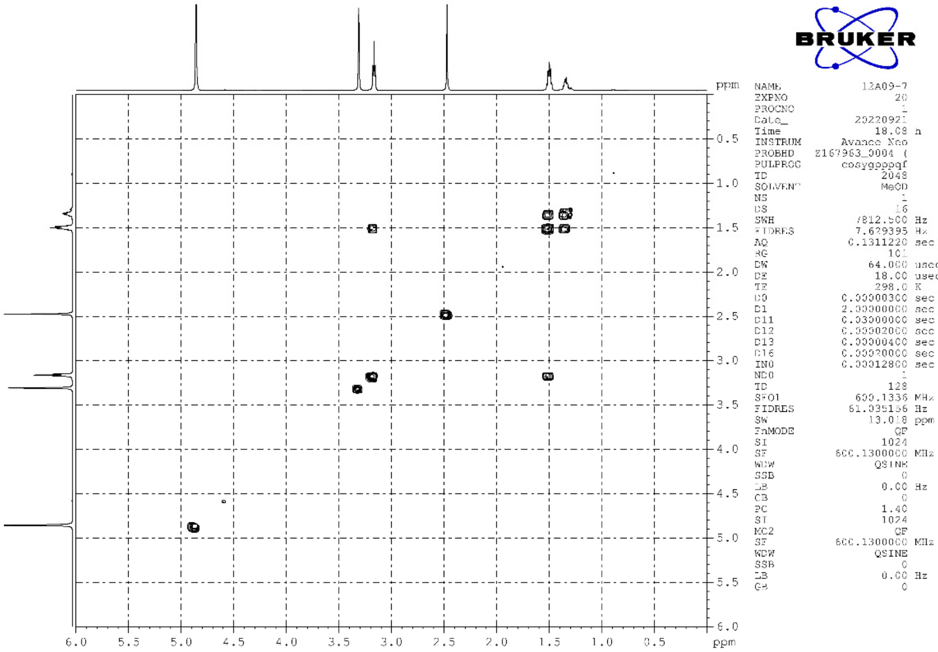

Figure S10. ^{1}H-^{1}H COSY spectrum of **2**

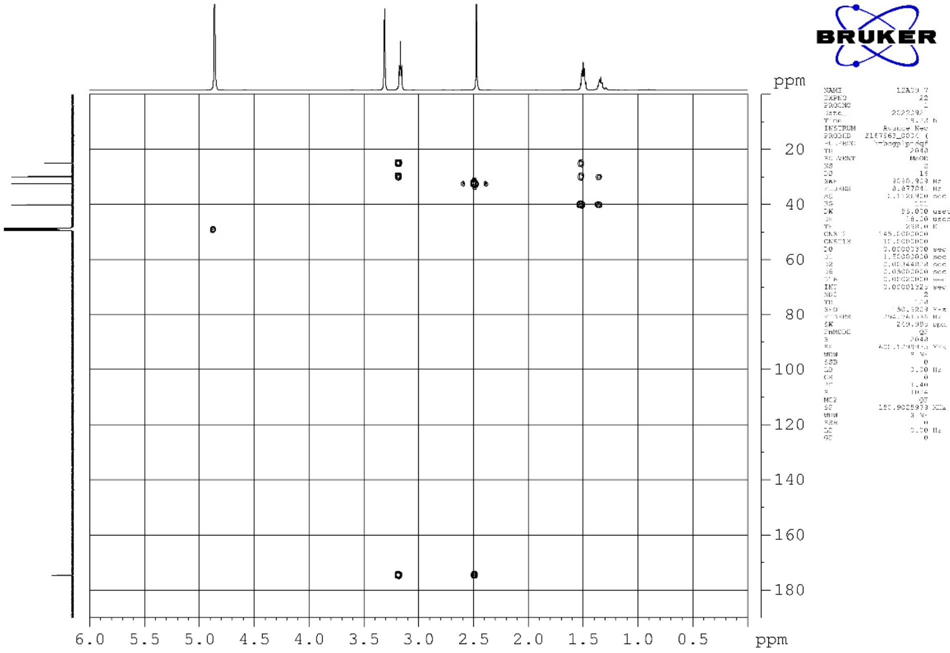

Figure S11. HMBC spectrum of **2**

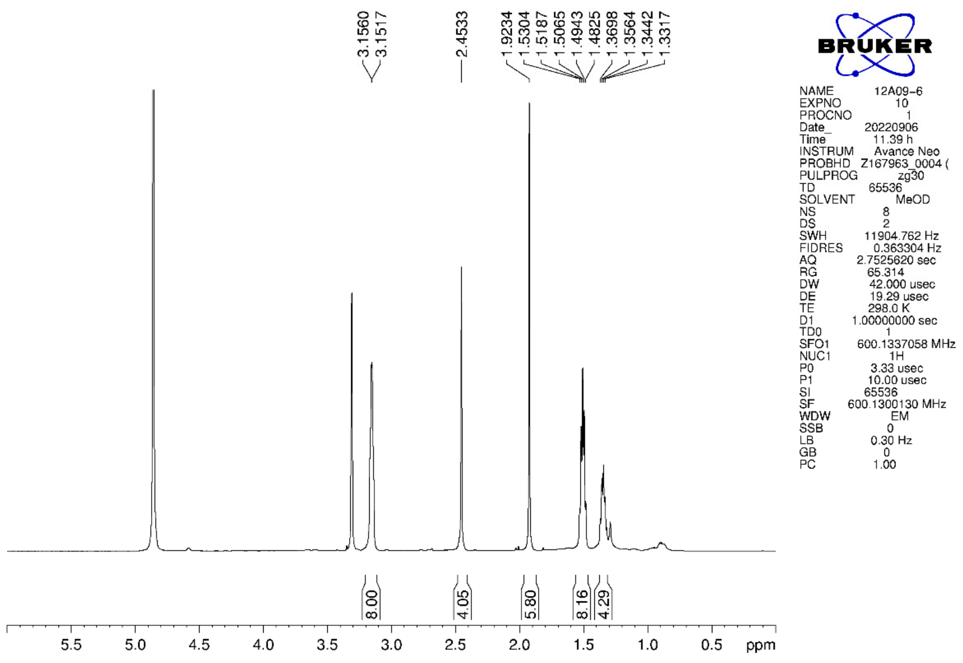

Figure S12. ^{1}H NMR (600 MHz, CD_3OD-d_4) spectrum of **3**

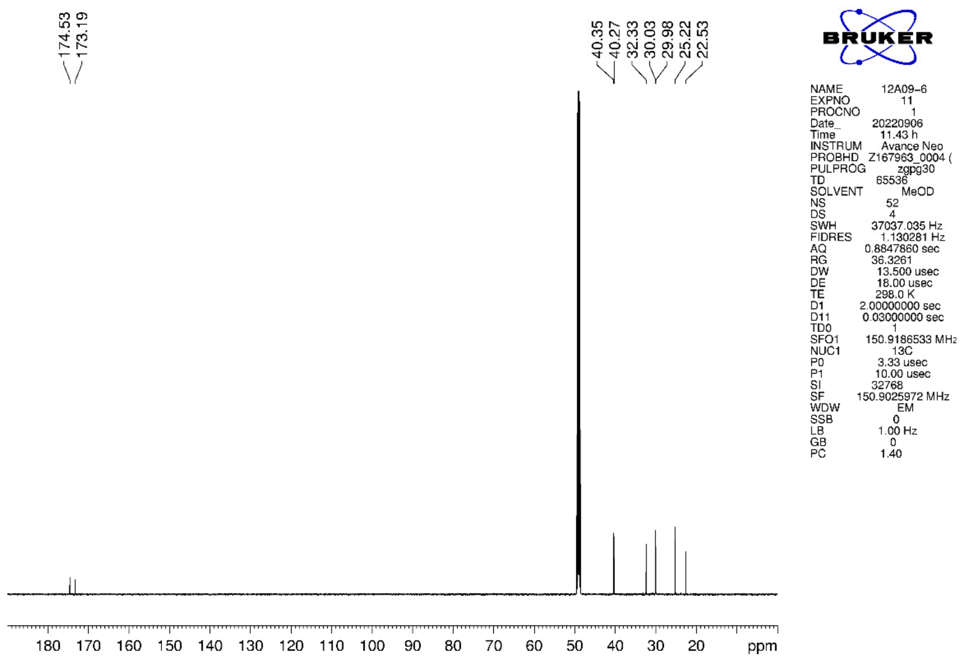

Figure S13. ^{13}C NMR (150 MHz, CD_3OD-d_4) spectrum of **3**

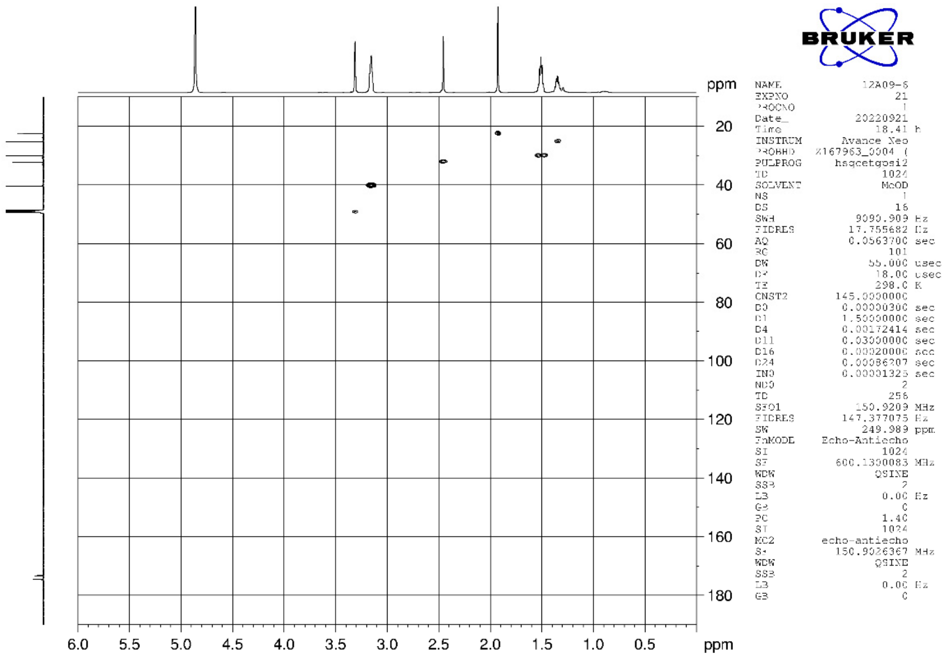

Figure S14. HSQC spectrum of **3**

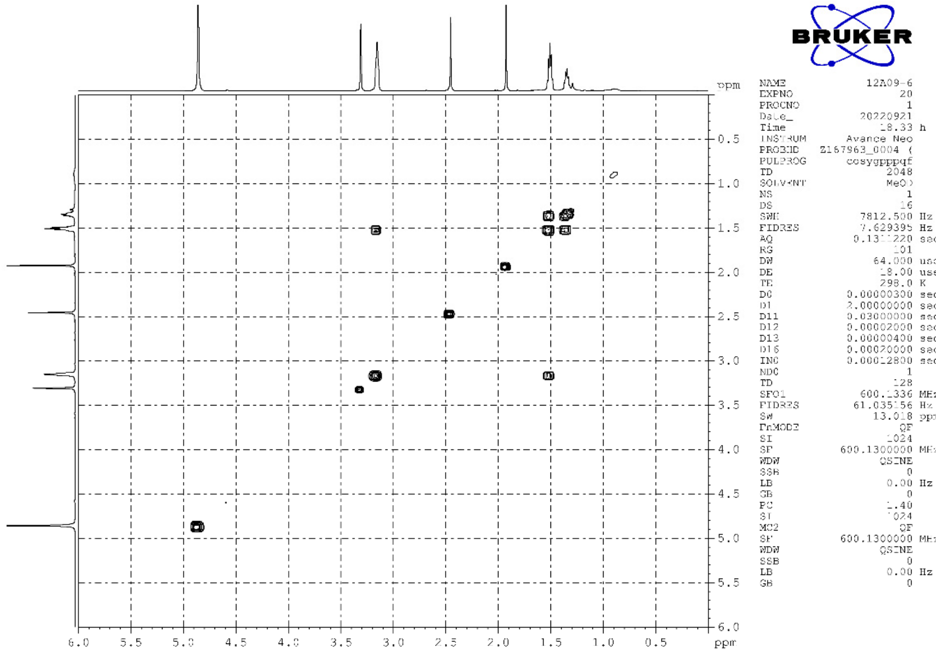

Figure S15. ^{1}H-^{1}H COSY spectrum of **3**

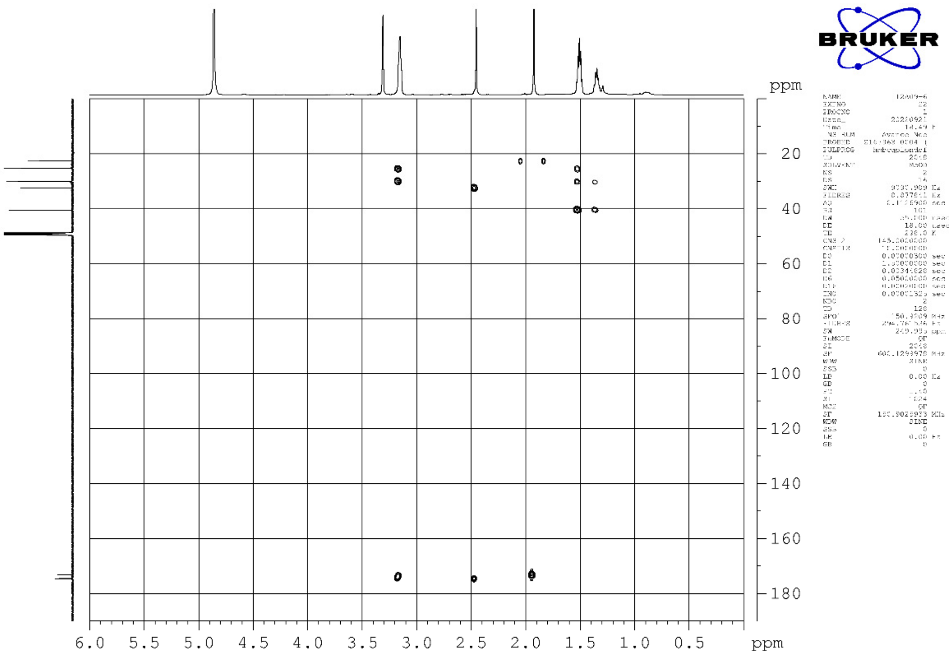

Figure S16. HMBC spectrum of **3**

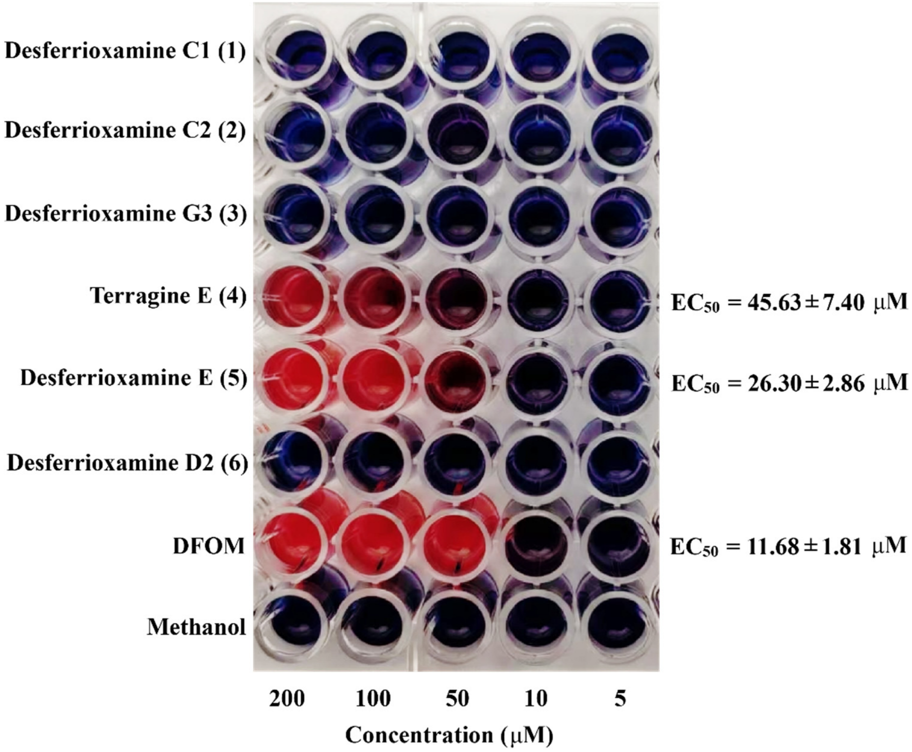

Figure S17. CAS assay results of **1-6** and DFOM. Concentration-dependent CAS liquid well-plate assay and EC_{50} values

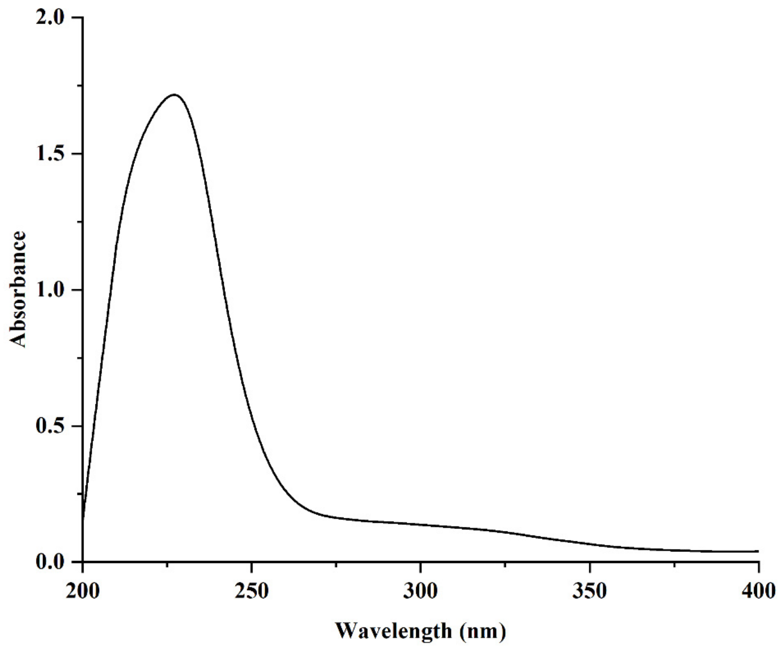

Figure S18. UV spectrum of **1**

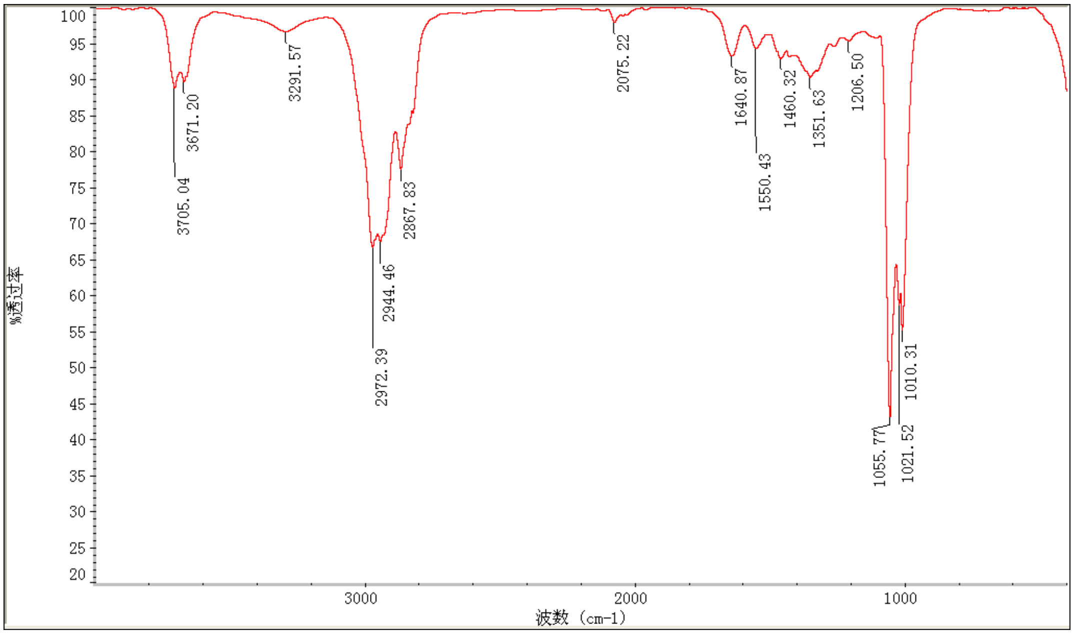

Figure S19. IR spectrum of **1**

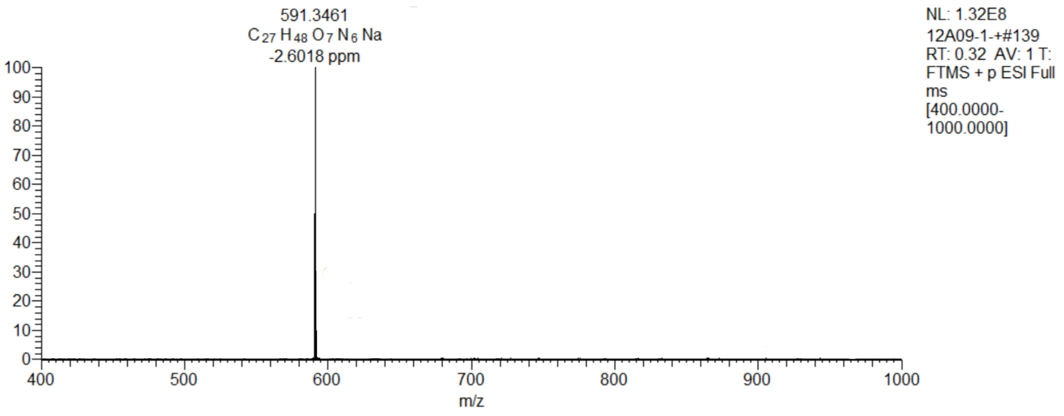

Figure S20. (+)-HRESIMS spectrum of **1**

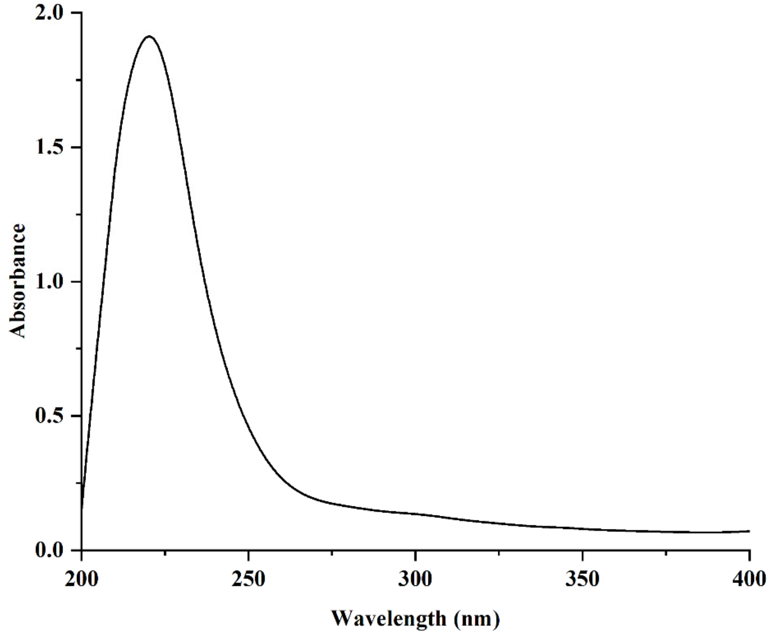

Figure S21. UV spectrum of **2**

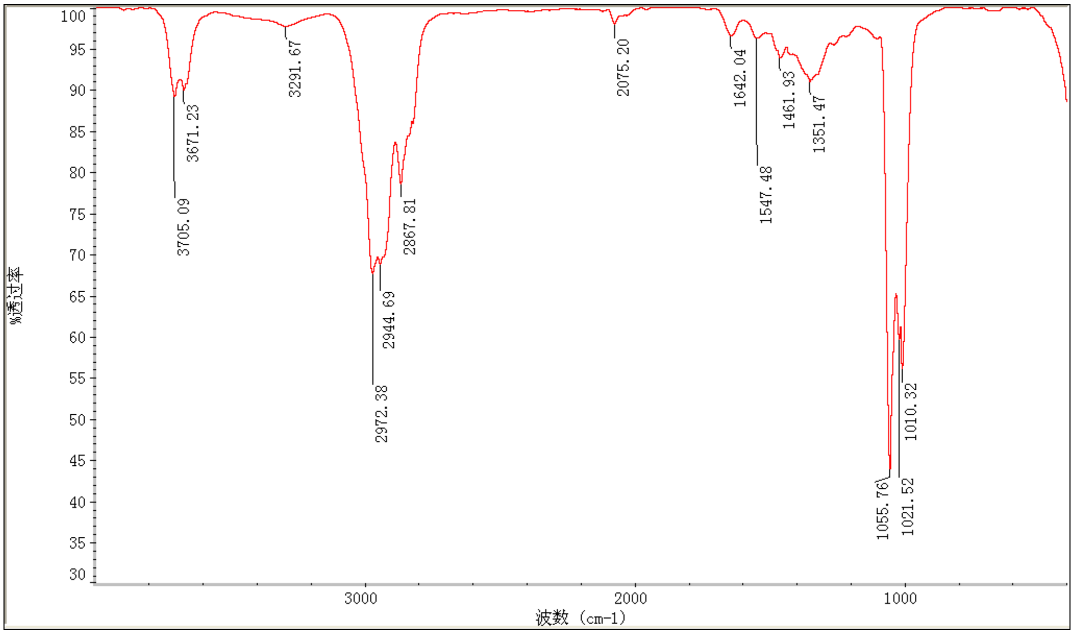

Figure S22. IR spectrum of **2**

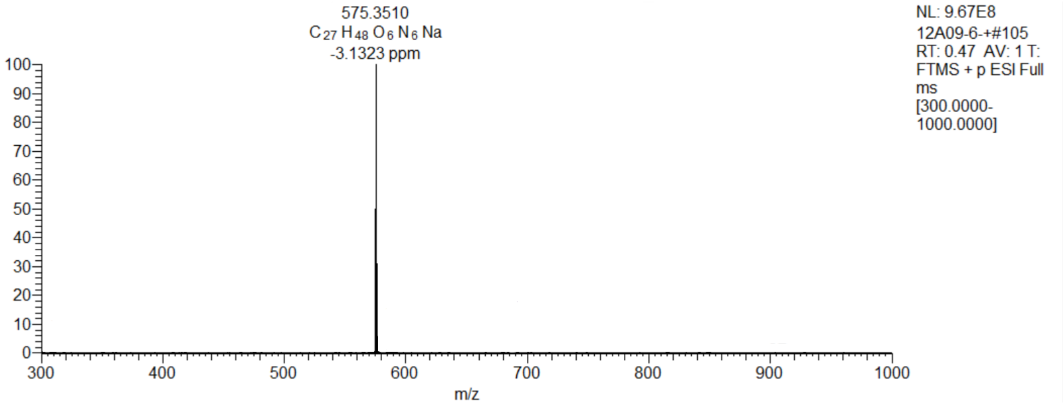

Figure S23. (+)-HRESIMS spectrum of **2**

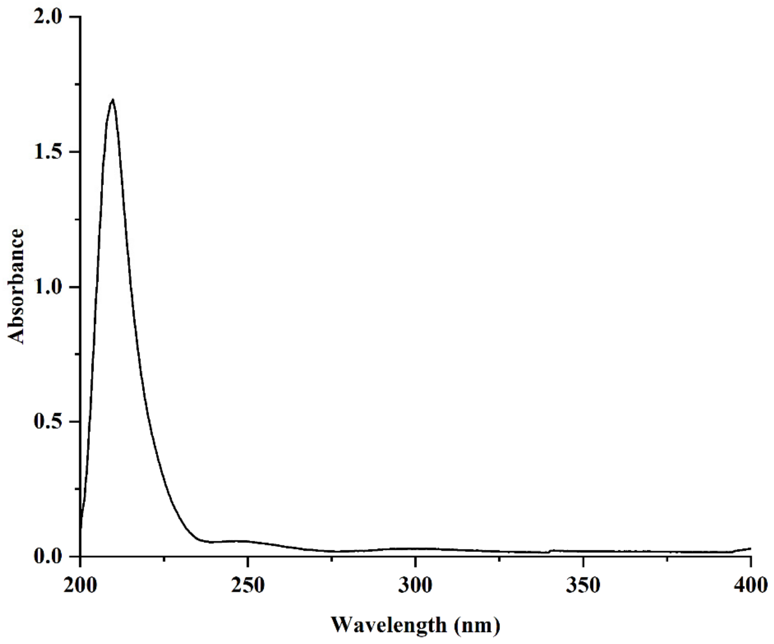

Figure S24. UV spectrum of **3**

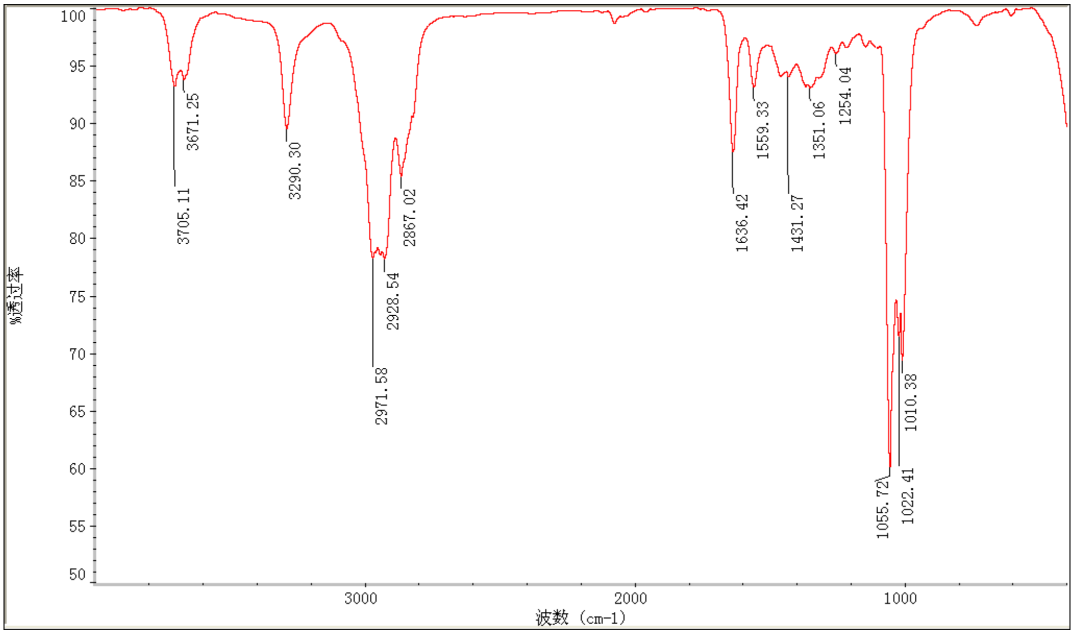

Figure S25. IR spectrum of **3**

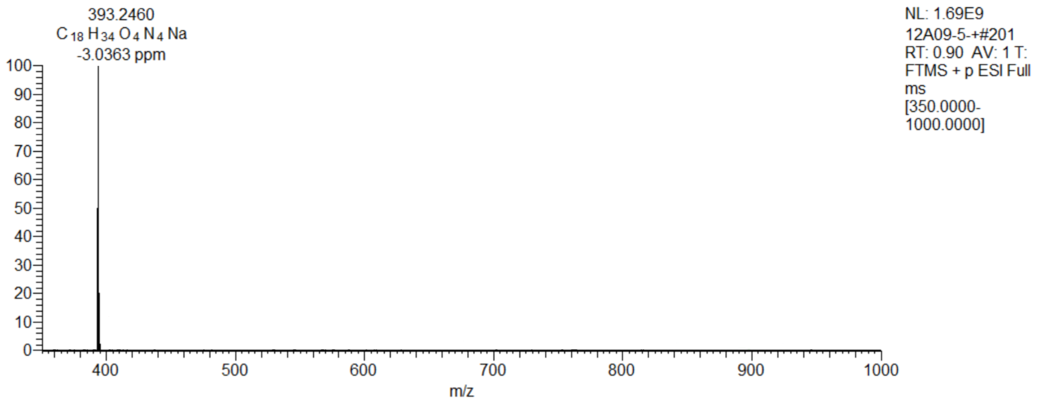

Figure S26. (+)-HRESIMS spectrum of **3**

Table S1 Number of genes associated with the 19 general COG functional categories

Type	Description of functional categories	Code	Value	Ratio
Information storage and processing	Translation, ribosomal structure and biogenesis	J	185	2.98%
	Transcription	K	609	9.82%
	Replication, recombination and repair	L	185	2.98%
Cellular processes and signaling	Cell cycle control, cell division, chromosome partitioning	D	32	0.52%
	Cell wall/membrane/envelope biogenesis	M	193	3.11%
	Cell motility	N	1	0.02%
	Posttranslational modification, protein turnover, chaperones	O	127	2.05%
	Intracellular trafficking, secretion, and vesicular transport	U	37	0.60%
Metabolism	Energy production and conversion	C	283	4.56%
	Amino acid transport and metabolism	E	315	5.08%
	Nucleotide transport and metabolism	F	102	1.65%
	Carbohydrate transport and metabolism	G	348	5.61%
	Coenzyme transport and metabolism	H	176	2.84%
	Lipid transport and metabolism	I	152	2.45%
	Inorganic ion transport and metabolism	P	297	4.79%
	Secondary metabolites biosynthesis, transport and catabolism	Q	129	2.08%
	Signal transduction mechanisms	T	271	4.37%
	Defense mechanisms	V	122	1.97%
Poorly characterized	Function unknown	S	1819	29.34%

Table S2 ^{1}H and ^{13}C NMR Data for 1 and 5 in DMSO-d_6 (600/150 MHz, δ in ppm)

Position	**1**		**5**	
	δ_H (J inHz)	δ_C,Type	δ_H (J inHz)	δ_C,Type
1		171.1 C		171.5 C
2	2.26 m	29.9 CH_2	2.27 m	30.0 CH_2
3	2.58 m	27.5 CH_2	2.57 m	27.5 CH_2
4		172.1 C		172.0 C
5	3.45 m	46.8 CH_2	3.45 m	46.8 CH_2
6	1.48 m	25.8 CH_2	1.48 m	25.8 CH_2
7	1.20 m	23.1 CH_2	1.19 m	23.2 CH_2
8	1.34 m	28.7 CH_2	1.36 m	28.6 CH_2
9	2.98 m	38.2 CH_2	2.98 m	38.3 CH_2
10		171.2 C		171.5 C
11	2.26 m	31.0 CH_2	2.27 m	30.0 CH_2
12	2.26 m	31.0 CH_2	2.57 m	27.5 CH_2
13		171.4 C		172.0 C
14	2.98 m	38.3 CH_2	3.45 m	46.8 CH_2
15	1.34 m	28.7 CH_2	1.48 m	25.8 CH_2
16	1.20 m	23.5 CH_2	1.19 m	23.2 CH_2
17	1.34 m	28.7 CH_2	1.36 m	28.6 CH_2
18	2.98 m	38.2 CH_2	2.98 m	38.3 CH_2
19		171.3 C		171.5 C
20	2.26 m	31.0 CH_2	2.27 m	30.0 CH_2
21	2.26 m	31.0 CH_2	2.57 m	27.5 CH_2
22		171.3 C		172.0 C
23	2.98 m	38.3 CH_2	3.45 m	46.8 CH_2
24	1.34 m	28.7 CH_2	1.48 m	25.8 CH_2
25	1.20 m	23.5 CH_2	1.19 m	23.2 CH_2
26	1.34 m	28.7 CH_2	1.36 m	28.6 CH_2
27	2.98 m	38.2 CH_2	2.98 m	38.3 CH_2
-NH	7.72 s		7.71 s	
N-OH	9.58 s		9.59 s	

Table S3 ^{1}H and ^{13}C NMR Data for **4** and terragine E in DMSO-d_6 (600/150 MHz, δ in ppm) [22]

Position	**4**		terragine E	
	δ_H (J inHz)	δ_C,Type	δ_H (J inHz)	δ_C,Type
1		171.3 C		171.3 C
2	2.26 m	30.0 CH_2	2.26 t (7.0)	29.9 CH_2
3	2.58 m	27.5 CH_2	2.57 t (7.0)	27.5 CH_2
4		172.0 C		172.0 C
5	3.45 m	46.8 CH_2	3.45 t (7.0)	46.8 CH_2
6	1.48 m	25.8 CH_2	1.47 p (7.0)	25.8 CH_2
7	1.20 m	23.1 CH_2	1.20 p (7.0)	23.1 CH_2
8	1.34 m	28.7 CH_2	1.34 p (7.0)	28.5 CH_2
9	2.98 m	38.3 CH_2	2.98 dt (6.0, 7.0)	38.2 CH_2
10		171.3 C		171.3 C
11	2.26 m	30.0 CH_2	2.26 t (7.0)	29.9 CH_2
12	2.58 m	27.5 CH_2	2.57 t (7.0)	27.5 CH_2
13		172.0 C		172.0 C
14	3.45 m	46.8 CH_2	3.45 t (7.0)	46.8 CH_2
15	1.48 m	25.8 CH_2	1.47 p (7.0)	25.8 CH_2
16	1.20 m	23.1 CH_2	1.20 p (7.0)	23.1 CH_2
17	1.34 m	28.7 CH_2	1.34 p (7.0)	28.5 CH_2
18	2.98 m	38.3 CH_2	2.98 dt (6.0, 7.0)	38.2 CH_2
19		171.4 C		171.4 C
20	2.26 m	31.1 CH_2	2.26 s	31.0 CH_2
21	2.26 m	31.1 CH_2	2.26 s	31.0 CH_2
22		171.4 C		171.4 C
23	2.98 m	38.3 CH_2	2.98 dt (6.0, 7.0)	38.2 CH_2
24	1.34 m	28.7 CH_2	1.34 p (7.0)	28.5 CH_2
25	1.20 m	23.5 CH_2	1.20 p (7.0)	23.4 CH_2
26	1.34 m	28.7 CH_2	1.34 p (7.0)	28.5 CH_2
27	2.98 m	38.3 CH_2	2.98 dt (6.0, 7.0)	38.2 CH_2
-NH	7.72 s		7.73 s	
N-OH	9.58 s		9.61 s	

Table S4 ^{1}H and ^{13}C NMR Data for **5** and desferrioxamine E in DMSO-d_6 (600/150 MHz, δ in ppm) [21]

Position	**5**		desferrioxamine E	
	δ_H (J inHz)	δ_C,Type	δ_H (J inHz)	δ_C,Type
1		171.5 C		171.8 C
2	2.27 m	30.0 CH_2	2.27 m	31.3 CH_2
3	2.57 m	27.5 CH_2	2.57 m	27.8 CH_2
4		172.0 C		172.3 C
5	3.45 m	46.8 CH_2	3.46 m	47.1 CH_2
6	1.48 m	25.8 CH_2	1.46 m	26.0 CH_2
7	1.19 m	23.2 CH_2	1.19 m	23.4 CH_2
8	1.36 m	28.6 CH_2	1.36 m	28.8 CH_2
9	2.98 m	38.3 CH_2	2.98 m	38.6 CH_2
10		171.5 C		171.8 C
11	2.27 m	30.0 CH_2	2.27 m	31.3 CH_2
12	2.57 m	27.5 CH_2	2.57 m	27.8 CH_2
13		172.0 C		172.3 C
14	3.45 m	46.8 CH_2	3.46 m	47.1 CH_2
15	1.48 m	25.8 CH_2	1.46 m	26.0 CH_2
16	1.19 m	23.2 CH_2	1.19 m	23.4 CH_2
17	1.36 m	28.6 CH_2	1.36 m	28.8 CH_2
18	2.98 m	38.3 CH_2	2.98 m	38.6 CH_2
19		171.5 C		171.8 C
20	2.27 m	30.0 CH_2	2.27 m	31.3 CH_2
21	2.57 m	27.5 CH_2	2.57 m	27.8 CH_2
22		172.0 C		172.3 C
23	3.45 m	46.8 CH_2	3.46 m	47.1 CH_2
24	1.48 m	25.8 CH_2	1.46 m	26.0 CH_2
25	1.19 m	23.2 CH_2	1.19 m	23.4 CH_2
26	1.36 m	28.6 CH_2	1.36 m	28.8 CH_2
27	2.98 m	38.3 CH_2	2.98 m	38.6 CH_2
-NH	7.71 s		7.70 s	
N-OH	9.59 s		9.73 s	

Table S5 ^{1}H and ^{13}C NMR Data for **6** and desferrioxamine D2 [23]

Position	**6***		desferrioxamine D2#	
	δ_H (J inHz)	δ_C,Type	δ_H (J inHz)	δ_C,Type
1		171.5 C		174.6 C
2	2.27 m	30.0 CH_2	2.46 t (6.8)	31.7 CH_2
3	2.57 m	27.5 CH_2	2.77 t (6.8)	29.0 CH_2
4		172.0 C		175.0 C
5	3.45 m	46.8 CH_2	3.60 t (6.3)	48.6 CH_2
6	1.48 m	25.8 CH_2	1.63 tt (7.3, 6.3)	27.2 CH_2
7	1.19 m	23.1 CH_2	1.32 m	24.5 CH_2
8	1.36 m	28.6 CH_2	1.51 m	29.7 CH_2
9	2.98 m	38.3 CH_2	3.17 t (6.3)	40.0 CH_2
10		171.5 C		174.6 C
11	2.27 m	30.0 CH_2	2.46 t (6.8)	31.7 CH_2
12	2.57 m	27.5 CH_2	2.77 t (6.8)	29.0 CH_2
13		172.0 C		175.0 C
14	3.45 m	46.8 CH_2	3.60 t (6.3)	48.6 CH_2
15	1.48 m	25.8 CH_2	1.63 tt (7.3, 6.3)	27.2 CH_2
16	1.19 m	23.1 CH_2	1.32 m	24.5 CH_2
17	1.36 m	28.6 CH_2	1.51 m	29.7 CH_2
18	2.98 m	38.3 CH_2	3.17 t (6.3)	40.0 CH_2
19		171.5 C		174.6 C
20	2.27 m	30.0 CH_2	2.46 t (6.8)	31.7 CH_2
21	2.57 m	27.5 CH_2	2.77 t (6.8)	29.0 CH_2
22		172.0 C		175.0 C
23	3.45 m	46.8 CH_2	3.60 t (6.3)	48.6 CH_2
24	1.48 m	25.8 CH_2	1.63 tt (7.3, 6.3)	25.0 CH_2
25	1.36 m	28.6 CH_2	1.51 m	27.4 CH_2
26	2.98 m	38.3 CH_2	3.17 t (6.3)	40.0 CH_2
-NH	7.71 s			
N-OH	9.59 s			

* Measured in DMSO-d_6. # Measured in CD_3OD-d_4.

References

1. Pan, H.Q. Design and prospects of an efficient mining pipeline for microbial natural products in the post-genome era. *J. Microbiol.* **2022**, *42*, 1–14.
